# A Bioinspired Membrane with Ultrahigh Li^+^/Na^+^ and Li^+^/K^+^ Separations Enables Direct Lithium Extraction from Brine

**DOI:** 10.1002/advs.202402898

**Published:** 2024-07-19

**Authors:** Faying Fan, Yongwen Ren, Shu Zhang, Zhilei Tang, Jia Wang, Xiaolei Han, Yuanyuan Yang, Guoli Lu, Yaojian Zhang, Lin Chen, Zhe Wang, Kewei Zhang, Jun Gao, Jingwen Zhao, Guanglei Cui, Bo Tang

**Affiliations:** ^1^ Qingdao Industrial Energy Storage Research Institute Qingdao Institute of Bioenergy and Bioprocess Technology Chinese Academy of Sciences Qingdao 266101 China; ^2^ Shandong Energy Institute Qingdao 266101 China; ^3^ Qingdao New Energy Shandong Laboratory Qingdao 266101 China; ^4^ Qingdao University Qingdao 266071 China; ^5^ Tang Bo's institution Laoshan Laboratory Qingdao China

**Keywords:** ion separation, lithium extraction, membrane, monovalent cation separation

## Abstract

Membranes with precise Li^+^/Na^+^ and Li^+^/K^+^ separations are imperative for lithium extraction from brine to address the lithium supply shortage. However, achieving this goal remains a daunting challenge due to the similar valence, chemical properties, and subtle atomic‐scale distinctions among these monovalent cations. Herein, inspired by the strict size‐sieving effect of biological ion channels, a membrane is presented based on nonporous crystalline materials featuring structurally rigid, dimensionally confined, and long‐range ordered ion channels that exclusively permeate naked Li^+^ but block Na^+^ and K^+^. This naked‐Li^+^‐sieving behavior not only enables unprecedented Li^+^/Na^+^ and Li^+^/K^+^ selectivities up to 2707.4 and 5109.8, respectively, even surpassing the state‐of‐the‐art membranes by at least two orders of magnitude, but also demonstrates impressive Li^+^/Mg^2+^ and Li^+^/Ca^2+^ separation capabilities. Moreover, this bioinspired membrane has to be utilized for creating a one‐step lithium extraction strategy from natural brines rich in Na^+^, K^+^, and Mg^2+^ without utilizing chemicals or creating solid waste, and it simultaneously produces hydrogen. This research has proposed a new type of ion‐sieving membrane and also provides an envisioning of the design paradigm and development of advanced membranes, ion separation, and lithium extraction.

## Graphical abstract

Inspired by the biological ion channel, a nonporous crystalline material‐based membrane with a rigidity‐confined ion channel is fabricated. It empowers excellent Li^+^/Na^+^, Li^+^/K^+^, Li^+^/Mg^2+^, and Li^+^/Ca^2+^ separations and enables a one‐step lithium extraction strategy from natural brines rich in Na^+^, K^+^, and Mg^2+^ without utilizing chemicals or creating solid waste.

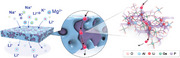

## 1 Introduction

Lithium‐ion batteries facilitate the energy revolution but the recent scarcity of continuous and sufficient lithium supply severely restricts their further implementation. Continental brines that come from salt lakes, oilfields, and concentrated seawater are the most common lithium resource on Earth^[^ ^1^, ^2^, ^3^, ^4^ ^]^; efficient and sustainable lithium extraction from natural brines is crucial for meeting the soaring lithium demand but remains a great challenge due to the composition complexity in brines.^[^ ^1^, ^3^, ^5^ ^]^ Recently, many powerful approaches including the adsorption method,^[^ ^6^, ^7^ ^]^ capacitive deionization (CDI),^[^ ^8^, ^9^ ^]^ liquid extraction,^[^ ^10^ ^]^ and membrane technology^[^ ^11^, ^12^ ^]^ have been developed to capture lithium from salt lake brine. Among them, membrane technology offers a more sustainable alternative, owing to these advantages of low carbon footprint, energy efficiency, small spatial requirements, and high efficiency—and is becoming one of the most used toolboxes in lithium extraction.^[^ ^13^, ^14^, ^15^ ^]^ Membranes made of polymers,^[^ ^11^ ^]^ graphene oxide (GO),^[^ ^16^, ^17^ ^]^ metal‐organic frameworks (MOFs),^[^ ^17^, ^18^ ^]^ covalent organic framework (COF),^[^ ^14^ ^]^ and MXenes^[^ ^19^ ^]^ have shown excellent Li^+^/Mg^2+^ separation based on a combination of Donnan exclusion, steric hindrance, and dielectric effects^[^ ^17^, ^20^, ^21^, ^22^, ^23^, ^24^ ^]^; however, these membranes shows poor Li^+^/Na^+^ and Li^+^/K^+^ separations,^[^ ^24^, ^25^ ^]^ and multistep, time‐consuming, and energy‐intensive pretreatment, e.g., solar‐evaporation, is employed to improve the brine grade which imposes practically inevitable limitations involving long production periods (more than 10 months), low lithium recovery, and heavy environmental pollution.

Membranes with the capability to separate Li^+^ not only from Mg^2+^ but also from Na^+^ and K^+^ are the determinant for extracting lithium from brines in a fast and sustainable way. Despite notable recent advances in Li^+^/Mg^2+^ separation^[^ ^20^, ^21^, ^26^, ^27^ ^]^; however, these membranes have struggled for efficient Li^+^/Na^+^ and Li^+^/K^+^ separation.^[^ ^24^, ^25^ ^]^ Indeed, the rational design of materials capable of precisely differentiating these monovalent cations has been a daunting challenge for several decades, primarily because these cations possess identical valence and only angstrom‐level radius difference.^[^ ^28^, ^29^, ^30^, ^31^, ^32^, ^33^ ^]^ Noteworthily, the challenge is also highlighted by the fact that K^+^ and Na^+^ have smaller radii and lower dehydration energies than those of Li^+^, resulting in much easier transmembrane diffusion for the formers in routine membranes.^[^ ^28^, ^34^ ^]^ Till now, only a few polystyrene sulfonates (PSS)‐inclusion membranes have exhibited moderate Li^+^/Na^+^ or Li^+^/K^+^ separation due to the specific binding affinities between cations and sulfonates in the channel;^[^ ^19^, ^35^ ^]^ however, their selectivities are limited in 35, which fall short of satisfactory for the practical application of direct lithium extraction from brines.

Biological ion channels with precise single‐ion selectivity play fundamental roles in regulating many physiological processes that ensure the normal functioning of living organisms.^[^ ^36^ ^]^ In the KcsA channel, a narrow segment is an ion‐selective filter exhibiting fast permeance toward dehydrated K^+^ ions but strictly rejecting Na^+^ ions, because Na^+^ possesses a larger hydrated diameter and dehydration energy than K^+^, endowing ultrahigh K^+^/Na^+^ selectivity of 10 000.^[^ ^37^ ^]^ The epithelial sodium channel contains a conserved tract with a pore radius comparable to the bare Na^+^ but much smaller than that of K^+^, which favors the preferred permeation of Na^+^ over K^+^ from both energy‐ and size‐matching perspectives, securing Na^+^/K^+^ permselectivity of 100–500.^[^ ^38^, ^39^ ^]^ Consequently, this size‐sieving principle inspired the exploration of artificial ion channels with appreciable selectivities by tailoring channel dimensions to well match targeted ions.^[^ ^31^, ^40^, ^41^, ^42^ ^]^ For instance, membranes derived from crown ethers have demonstrated remarkable Li^+^/Na^+^ selectivity due to the size‐matching complexation between the 14‐crown‐4 and Li^+^.^[^ ^43^, ^44^ ^]^ However, their selectivity toward Li^+^/Na^+^ and Li^+^/K^+^ is restricted to only 24.6 and 21.3, respectively, owing to the intrinsic flexibility and disorder of the channel structure.^[^ ^45^ ^]^

Herein, we draw inspiration from single‐species separation behavior in natural biological ion channels and present a membrane featuring rigidity‐confined ion channels that snugly accommodate naked Li^+^ ions but block other competing cations, empowering synchronously ultrahigh Li^+^/Na^+^, Li^+^/K^+^, Li^+^/Mg^2+^, and Li^+^/Ca^2+^ separation selectivities along with ultrafast Li^+^ transport. This membrane, with the chemical and structural stability in harsh natural aqueous operation environments, enables a continuous, direct, and one‐step lithium extraction even from low‐grade brine simultaneously rich in Na^+^, K^+^, Mg^2+^, and Ca^2+^. This ion‐sieving mechanism has been further proposed with the assistance of DFT calculation. The intrinsic differences of overall transmembrane obstacles consisting of dehydration and bulk diffusion energy barriers contribute to this unusual ion‐sieving behavior, which endows an important milestone of single‐species separation.

## 2 Results and Discussion

### 2.1 Design and Fabrication of the Bioinspired Single‐Li^+^ Transport Membrane

**Figure 1:**
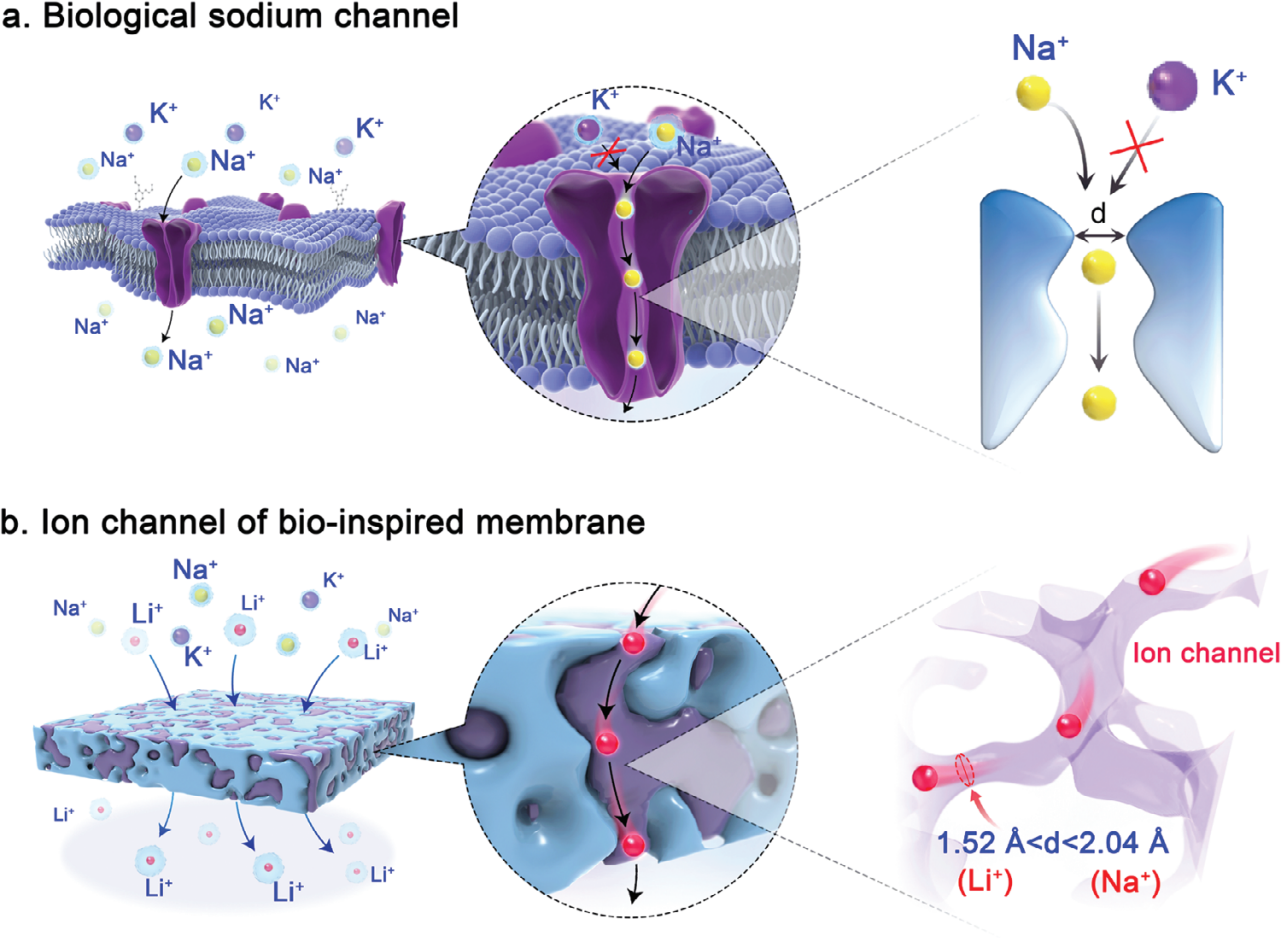
Schematic depiction of membrane and cation transport details. a) Biological Na^+^ channel, permeating naked Na^+^ but blocking K^+^. b) Ideal Li^+^ channel of bio‐inspired membrane for Li^+^/Na^+^ and Li^+^/K^+^ separations; the channel size is between the diameters of Li^+^ (1.52 Å) and Na^+^ (2.04 Å), which only permeates naked Li^+^ while blocking other competing cations.

In the biological Na^+^ channel, narrow tunnel‐like membrane proteins with negatively charged terminals decorated along the channel selectively allow the less hydrated Na^+^ to permeate, but block K^+^ because the channel size is even smaller than the diameter of naked K^+^ (**Figure** **1**a).^[^ ^38^, ^39^ ^]^ Intuitively, by regulating the ion channel size to accommodate the naked Li^+^ ion (with a diameter of 1.52 Å) while blocking the larger naked Na^+^ ion (with a diameter of 2.04 Å), selective permeation can be achieved to the naked Li^+^ ion, enabling effective Li^+^/Na^+^ and Li^+^/K^+^ separations (Figure 1b). However, currently reported membrane materials, including MOFs, COFs, two‐dimensional (2D) materials, and polymers, pose challenges in meeting this requirement. The ion channels in these materials are significantly larger than the required dimensions.^[^ ^46^, ^47^ ^]^ Additionally, the thermal motion and swelling of the non‐rigid or semi‐rigid polymer segments are prone to facilitate ion diffusion but also cause poor selectivity.^[^ ^48^ ^]^ Certainly, it is imperative to investigate innovative materials featuring ion channels that satisfy these size requirements and exhibit rigidity to emulate the single‐ion separation behavior based on the size‐sieving effect observed in biological ion channels, to achieve precise separations of Li^+^/Na^+^ and Li^+^/K^+^.

**Figure 2:**
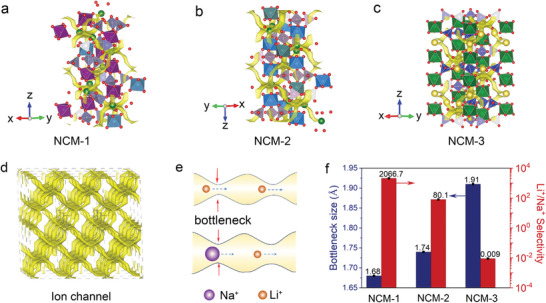
Crystal structure and Li^+^/Na^+^ selectivity of the ceramic membranes. Crystal structure of the bioinspired membrane: a) NCM‐1, b) NCM‐2, and c) NCM‐3, the ion channels were marked with yellow; d) the long‐range ordered and continuous ion channels in 4 × 3 × 1 supercell of the NCM‐1 membrane; e) cations diffuse in ion channels with different bottleneck; f) the bottleneck size and Li^+^/Na^+^ selectivity of the membranes.

NASICON‐type materials are nonporous crystalline materials (NCM) with the chemical structure of *A* *_x_* *M* *_y_* (*B*O_4_)_3_, consisting of corner‐sharing *M*O_6_ octahedra and *B*O_4_ tetrahedra that assemble to form a robust 3D network structure. A sub‐nano confined, rigid, and long‐range ordered ion channel, surrounded by oxygen atoms, is formed among the interstitial sites or cavities for mobile *A* ^+^ ions migration.^[^ ^49^, ^50^, ^51^, ^52^ ^]^ Although NASICON‐type materials are commonly used as electrodes and electrolytes for batteries; however, there have been rare reports that have systemic focused on the ion‐sieving performance. We have first calculated that the three typical NASICON‐type materials, Li_1.5_Al_0.5_Ge_1.5_(PO_4_)_3_ (NCM‐1), Li_1.5_Al_0.5_Ti_1.5_(PO_4_)_3_ (NCM‐2), and Na_2.5_Zr_2_Si_1.5_(PO_4_)_3_ (NCM‐3), which are constructed with corner‐sharing PO_4_/SiO_4_ tetrahedra and ZrO_6_/AlO_6_/TiO_6_ octahedra, individually possess the bottlenecks with the size of 1.68 Å, 1.74 Å, and 1.91 Å in their ion channel (**Figure** **2**a–c). All of them fall between that of the naked Li^+^ and naked Na^+^, and they can be regarded as potential models for ionic cut‐off filters in the separation of Li^+^/Na^+^ and Li^+^/K^+^ based on the naked ion‐sieving effect. To elucidate the ion channel more clearly, the yellow isosurfaces depicted in Figure 2a–c represent the simulated ion conduction pathway spread throughout the structure by calculating the probability density map of mobile ions using the BVpath calculation. The inherent long‐range ordered crystalline structure results in ion channels displaying regular and continuous characteristics, as illustrated in Figure 2d. This necessitates the continuous traversal of mobile ions through successive and periodic bottlenecks along the ion channel, which will intensify the ion‐sieving capability of the ion channel and improve the sieving selectivity of membranes.

Dense and compact membranes were prepared by isostatic pressing coupled with an annealing process to embody the ion‐sieving performance of these nonporous crystalline materials. The as‐prepared NCM‐1, NCM‐2, and NCM‐3 membranes are respectively well in accordance with the LiGe_2_(PO_4_)_3_ (PDF# 80‐1924), LiTi_2_(PO_4_)_3_ (PDF# 35‐0754), and Na_2.5_Zr_2_Si_1.5_(PO_4_)_3_ (PDF# 36‐0350),^[^ ^53^, ^54^ ^]^ according to the XRD pattern shown in Figures S1–S3 (Supporting Information). Through‐pores or other imperfections in the membranes have no ion separation selectivity which will reduce the ion separation selectivity of membranes since ions could transport through these imperfections rather than ion channels. Besides, these imperfections also impair the robustness of the membranes. Therefore, pore‐free, crack‐free, and compactness are very important for the selectivity of membranes.^[^ ^55^, ^56^, ^57^ ^]^ It is seen from the cross‐section SEM image (Figure S4, Supporting Information) that the micrometer‐sized grains are tightly packed and there are no cracks or continuous pores between them, and no through‐pores or cracks were seen on the NCM‐1 membrane even after ion‐beam cleaning. The diffusion coefficient of Li^+^ was measured by pulsed‐field gradient‐stimulated‐echo nuclear magnetic resonance (PFG‐NMR) experiments (Figure S5, Supporting Information). The calculated diffusion coefficient of NCM‐1 powders and membrane was 8.34x10^−11^ and 1.57x10^−10^ m^2^ s^−1^, respectively, indicating the good compactness and high intrinsic ion transport properties of the NCM‐1 membrane.

The Li^+^/Na^+^ selectivities of these membranes were first evaluated by a homemade device (Figure S6, Supporting Information), in which membranes were individually placed between the feed solution (a mixed solution of 0.1 m NaCl and 0.1 m LiCl) and permeate solutions (0.01 m HCl). The cations in the feed solution migrate through the membrane to the permeate solution under the electric driving, and the cation migration was determined by monitoring the variation of cation concentration in the permeate solution. Figure 2f shows that the Li^+^/Na^+^ selectivity of the NCM‐1 and NCM‐2 membranes reaches 2066.7 and 80.1, whereas that of the NCM‐3 membrane is as low as 0.009. These observations also suggest that a decrease in the bottleneck size of the ion channel leads to an increase in Li^+^/Na^+^ selectivity, highlighting the critical role of ion channel size in determining Li^+^/Na^+^ separation.

However, the NCM‐2 membrane is prone to breakage in NaCl solution only for few hours. The XRD pattern in Figure S7 (Supporting Information) shows that after being immersed in NaCl solution, part of the LiTi_2_(PO_4_)_3_ in NCM‐2 was transformed into NaTi_2_(PO_4_)_3_, demonstrating that the Na^+^ partially substituted Li^+^ in NCM‐2. The density functional theoretical (DFT) calculation results in Table S2 (Supporting Information) verify that the substitution results in lattice volume expansion due to the large radius of Na^+^, which is the main reason for the breakage of the NCM‐2 membrane in the Na^+^ solution. Conversely, the NCM‐1 shows no significant change when immersed in NaCl solution (shown in Figure S8, Supporting Information). Additionally, the DFT calculation results in Table S2 (Supporting Information) further prove the substitution of Li^+^ in NCM‐1 with Na^+^ is energy obligatory; however, the energy for the former three Na^+^ substitutions in NCM‐2 are negative, while the energy for the further substitutions is positive, indicating that the Na^+^ can spontaneously substitute part of Li^+^ in NCM‐2 but unable for the NCM‐1. Considering the excellent Li^+^/Na^+^ separation capability and good stability of NCM‐1 membrane, we will further investigate the performance of NCM‐1 membrane in detail to unveil the ion‐sieving characteristics of this new type of membrane.

### 2.2 Cation Selective Transport Performance of Membranes

**Figure 3:**
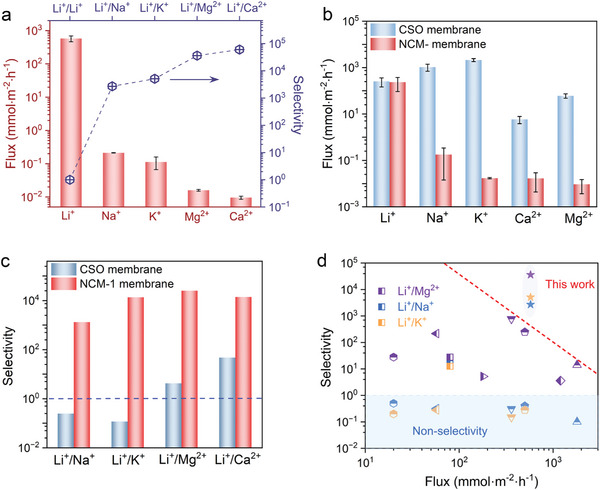
Cation sieving performance of membranes. a) Cation fluxes and selectivities of NCM‐1 membrane in the single‐salt systems. b,c) Cation fluxes and selectivities of NCM‐1 and CSO membranes in the mixed‐salt system. d) Comparison of the Li^+^/Na^+^, Li^+^/K^+^, and Li^+^/Mg^2+^ selectivity and Li^+^ flux values of various membranes (data of Li^+^/Mg^2+^, Li^+^/Na^+^, and Li^+^/K^+^ separations are shown in purple, blue, and yellow, respectively); The dashed line was added manually to show the trade‐off between the Li^+^ flux and selectivity based on the state‐of‐the‐art membranes. Error bars represent the standard deviation of three measurements of a sample.

The cation‐selective transport performance of the membrane was assessed in the single‐salt systems under an electric driving force of 3 V. The feed compartment was filled with 0.1 m LiCl, MgCl_2_, KCl, NaCl, or CaCl_2_ solution, respectively. The Li^+^ flux of the NCM‐1 membrane is 575.8 mmol m^−2^ h^−1^, whereas the fluxes of Na^+^, K^+^, Mg^2+^, and Ca^2+^ are almost several orders of magnitude lower than that of Li^+^ (**Figure** **3**a), revealing that this NCM‐1 membrane exhibits exceptional selectivity in transporting Li^+^ over these competition cations. Reaching Li^+^/Na^+^, Li^+^/K^+^, Li^+^/Mg^2+^, and Li^+^/Ca^2+^ selectivities as high as 2707.4, 5109.8, 36161.7, and 60269.6, respectively. This result confirms this membrane with the bioinspired “Li^+^ ion channel” can recognize the Li^+^ ions and selectively let them pass through, even comparable to those of biological ion channels.^[^ ^38^ ^]^

In natural brine, Li^+^ typically coexisted with varying concentrations of Na^+^, K^+^, Mg^2+^, and Ca^2+^. In this instance, the ion sieving performance conducted in a mixed‐salt system is typically of more practical significance, thus a mixed solution containing 0.1 m LiCl, MgCl_2_, KCl, NaCl, and CaCl_2_ was used as the feedstock to further investigate the ion separation performance. For the NCM‐1 membrane, although the mixed‐salts system shows almost 2 times decreased Li^+^ flux compared to the single‐salt system, the separation selectivities in the mixed‐salt system remain at very high levels with 1325.2, 13695.9, 25283.7, and 14048.2 for Li^+^/Na^+^, Li^+^/K^+^, Li^+^/Mg^2+^, and Li^+^/Ca^2+^, respectively. For comparison, a representative commercial monovalent selective cation exchange membrane (CSO membrane, GC Engineering Co., Ltd.) was also adopted to evaluate the cation separation performance. Consistent with reports,^[^ ^58^ ^]^ the CSO membrane shows good Li^+^/Mg^2+^ and Li^+^/Ca^2+^ separation performance; However, the fluxes of monovalent cations in the CSO membrane follow the orders of K^+^>Na^+^>Li^+^, and both Li^+^/Na^+^ and Li^+^/K^+^ selectivities are lower than 1, indicating the CSO membrane is incapable for Li^+^/Na^+^ and Li^+^/K^+^ separation (Figure 3c).

The challenge of Li^+^/Na^+^ and Li^+^/K^+^ separations has posed a persistent conundrum for both academic and industrial sectors over several decades.^[^ ^59^ ^]^ It is worth noting that only a small fraction of reported membranes demonstrates a subpar selectivity for Li^+^/K^+^ and Li^+^/Na^+^ separation (as shown in Figure 3d and Table S3, Supporting Information), whereas the majority of previously reported membranes are incapable of such separation (their selectivities are lower than 1).^[^ ^19^, ^28^, ^60^, ^61^, ^62^, ^63^, ^64^ ^]^ What was particularly intriguing, the NCM‐1 membrane in our work displays unprecedented Li^+^/K^+^ and Li^+^/Na^+^ selectivities, even surpassing the current state‐of‐the‐art membranes by several orders of magnitude. Additionally, the Li^+^/Mg^2+^ selectivity offered by the NCM‐1 membrane was almost orders of magnitude higher than the cutting‐edge membranes (e.g., MOFs and porous organic cages membranes^[^ ^28^, ^61^ ^]^). Moreover, it is widely accepted that membranes are typically subject to the well‐known permeability‐selectivity trade‐off, that is the high selectivity will sacrifice the permeability, and vice versa.^[^ ^57^ ^]^ In this regard, the results reported in this work are encouraging, considering that both the selectivity and permeability of the NCM‐1 membrane are located at the upper bounds in comparison.^[^ ^14^, ^19^, ^28^, ^61^, ^63^, ^64^ ^]^ Hence, this NCM‐1 membrane outperforms most conventional membranes made of polymeric materials, 2D materials, or porous crystalline materials in terms of Li^+^ selective transport performance, it motivates us to uncover the intrinsic ion‐sieving mechanism of this membrane.

### 2.3 Cation Sieving Mechanism in Ceramic Membrane

**Figure 4:**
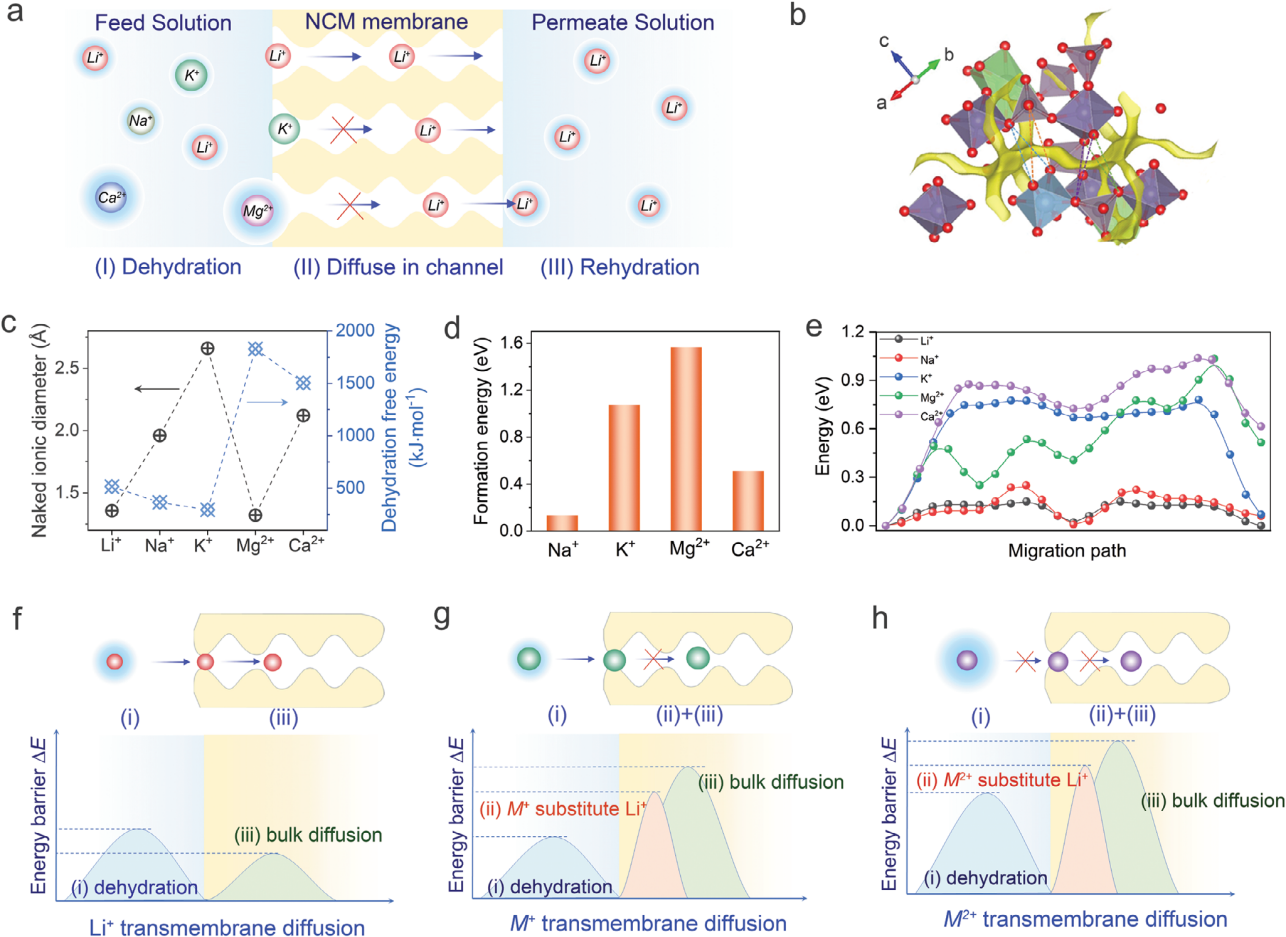
Cation‐sieving mechanism of the NCM‐1 membrane. a) Schematic of cations migrating from feed to permeate solution via three processes: dehydration, naked cation diffusion through membrane, and rehydration. b) Crystal structure of NCM‐1 membrane with a rhombohedral lattice and potential Li^+^ transport pathways across a unit cell in NCM‐1 (yellow channel for native Li^+^, and four bottlenecks marked by the dot lines). c) Naked ionic diameter and dehydration‐free energy of cations. d) The formation energy of substitution of Li^+^ sites with *M* *^n^* ^+^ in NCM‐1. e) Diffusion energy barriers for cation transport in bulk NCM‐1. f−h) Schematics of Li^+^, *M* ^+^ (Na^+^ and K^+^), and *M* ^2+^ (Mg^2+^ and Ca^2+^) transmembrane diffusion, and the energy barriers that include dehydration, substitution of Li^+^ site, and bulk diffusion need to breakthrough.

Uncovering the ion‐sieving mechanism underlying this bioinspired NCM‐1 membrane is crucial for achieving the possibility of designing Li^+^‐sieving membrane. Cations in the aqueous solution are in their hydrated state, in which naked cations are surrounded by water molecules, and the transport of cations from the feed solution to the permeate solution typically undergoes three steps^[^ ^65^, ^66^, ^67^ ^]^: i) dehydration of hydrated cations in the feed solution near the membrane surface, ii) naked cations diffuse in bulk NCM‐1, iii) naked cations rehydrate in the permeate solution (**Figure** **4**a). It is theoretically apparent that the former two steps are the determining factors for the selective transport of cations. Since the bottleneck size of the ion channels in NCM‐1 is much smaller than the diameters of hydrated metal cations but comparable to that of naked ones, hydrated cations must overcome energy barriers to strip off their hydration shell before entering the ion channel.^[^ ^22^ ^]^ The dehydration‐free energy for Li^+^, Na^+^, and K^+^ is 515, 365, and 271 kJ mol^−1^, respectively, whereas Ca^2+^ and Mg^2+^ have to consume nearly three times as much energy as Li^+^ (Figure 4c),^[^ ^68^ ^]^ contributing to the outstanding Li^+^/Mg^2+^ and Li^+^/Ca^2+^ separation.

Diffusion of dehydrated cations in ion channels is another crucial factor for cation separation, and the bond valence energy landscape (BVEL) method was applied to simulate cation diffusion pathways to investigate the cation diffusion behaviors in the ion channel. As shown in Figure 4b, these corner‐sharing octahedra and tetrahedra form the rigid structural skeleton for the channel, and Li^+^ acts as the mobile ions that can easily diffuse in the channel due to the electrostatic interaction. Although native Li^+^ ions can freely transport in the channels, rival *M* *^n^* ^+^ (*M* *^n^* ^+^ = Na^+^, K^+^, Mg^2+^, and Ca^2+^) cations are subjected to a substitution process of Li^+^ sites as a crucial pre‐requisite for further diffusion in the ion channel. We thus evaluated the possibility of *M* *^n^* ^+^ entering the NCM‐1 ion channel by calculating the formation energy of one *M* *^n^* ^+^ substituting for one Li^+^ in the NCM‐1,^[^ ^69^, ^70^ ^]^ and the calculation result revealed that all the substitutions will result in positive formation energy. This is attributed to the fact that the larger ionic radii of K^+^ and Na^+^ distort the crystal structure, while greater charge density of Mg^2+^ and Ca^2+^ induces a strong polarization effect on the surrounding atoms, and all of them will bring energy increasing in the crystal structure. Consequently, the endothermic and thermodynamically unfavorable nature of the substitution imposes an extra energy penalty hindering cation permeation (Figure 4d).^[^ ^71^, ^72^ ^]^

Once entering the channel, naked cations will diffuse in the channel via get through the periodic and long‐range ordered bottlenecks by overcoming continuous and regular energy barriers. We further calculated the diffusion energy barriers of Li^+^, Na^+^, K^+^, Mg^2+^, and Ca^2+^ in a unit cell via the knock‐off diffusion mechanism with cooperative interactions of at least two ions triggered by strong electrostatic forces. The profiles of diffusion energy barriers for cation transport in bulk NCM‐1 as depicted in Figure 4e demonstrate that *M* *^n^* ^+^ cations (*M* *^n^* ^+^ = Na^+^, K^+^, Mg^2+^, and Ca^2+^) need to breakthrough much more energy barriers to diffuse in the ion channels than Li^+^, which is most probably due to the following two reasons. I) Given the larger diameter of Na^+^ and K^+^ than the ion channel size of NCM‐1 (1.68 Å), the transport of Na^+^ and K^+^ will be impeded due to the size‐sieving effect.^[^ ^73^, ^74^ ^]^ Additionally, as illustrated in Figure 2e, it has been confirmed that a smaller bottleneck size results in greater Li^+^/Na^+^ selectivity, further affirming the significance of size effect in ion separation. II) The oxygen atoms of polyanionic moieties in ion channels exhibit greater affinity for divalent cations (Mg^2+^ and Ca^2+^) as compared to Li^+^. In other words, when Mg^2+^ and Ca^2+^ are introduced into the channel, they become firmly bound by the oxygen around the ion channel, resulting in a significantly higher diffusion energy barrier.^[^ ^49^, ^75^ ^]^

Based on the above discussions, we proposed a schematic mechanism for Li^+^ sieving from *M* *^n^* ^+^ by using the bioinspired NCM‐1 membrane (Figure 4f–h). The synchronously obtained ultrahigh Li^+^/M*^n^* ^+^ selectivities could be ascribed to discriminating sequential energy barriers involving dehydration of hydrated cations, and substitution of Li^+^ sites with M*^n^* ^+^ as well as cation bulk diffusion in the channels. Of note, for Mg^2+^ and Ca^2+^, all energy barriers encountered during the transmembrane transport are essentially larger than those of Li^+^ (Figure 4h). Regarding the hydrated Na^+^ and K^+^ ions, despite their hydration‐free energy being slightly lower than that of Li^+^, the confined and rigid ion channels create elevated and periodic energy barriers for the substitution of Li^+^ and their further bulk diffusion within the ion channel, rendering their permeation into and through the NCM‐1 membrane particularly challenging (Figure 4g). Noteworthy, the rigidity of the inorganic channel frameworks ensures the maintenance of the size‐matched effect during separation is crucial for this unprecedented Li^+^/Na^+^ and Li^+^/K^+^ separations. This finding is in line with a widely held understanding in the field of biological membranes that rigidifying the selectivity filter pores to optimally accommodate the target cations can improve permeance selectivity.

### 2.4 Robustness of Bioinspired NCM‐1 Membrane

It is established that membranes made of polymeric or 2D materials are susceptible to biological or chemical fouling, swelling in hygroscopic environments, and structural damage from continual exposure to chlorine or oxygen species.^[^ ^16^, ^27^, ^76^ ^]^ Each of these detriments will significantly deteriorate separation performance and increase energy consumption.^[^ ^27^, ^77^, ^78^ ^]^ We immersed the NCM‐1 membrane in water and a mixed salt solution (including 0.1 m LiCl, NaCl, KCl, MgCl_2_, and CaCl_2_) for 7 d, respectively. Good chemical and structural stability are indicated by the absence of apparent alterations in the crystal structure (Figure S14, Supporting Information). Likewise, the mixed‐salt contains high concentrations of chlorine. In addition, basic species (at the cathode), acidic species (at the anode), and powerfully oxidizing species (ClO^−^) were also produced during operation. Even in such harsh environments, our NCM‐1 membrane exhibits no discernible morphology (Figure S15, Supporting Information) or crystal structure alterations (Figure S16, Supporting Information), demonstrating chlorine‐resistant, acid‐resistant, base‐resistant, and anti‐oxidative properties. Such versatility empowers the NCM‐1 membrane substantial prerequisite in practical applications.

### 2.5 Lithium Extraction from Raw Brine

Directly extracting lithium from raw brine is still in its infancy since several orders of magnitude Na^+^, K^+^, Mg^2+^, and Ca^2+^ ions coexist with Li^+^ in raw brine; however, due to the lack of efficient Li^+^/Na^+^ and Li^+^/K^+^ separation strategy, a pretreatment so‐called solar evaporation is employed to improve the quality of raw brine in industrial lithium extraction. It involves the use of solar energy to evaporate water from the brine, which helps concentrate the lithium content and remove the Na^+^ and K^+^ in the brine. However, it has drawbacks such as prolonged production periods (10–24 months), high energy consumption, and significant amounts of solid waste. These limitations restrict the lithium production capacity and further worsen the already fragile ecological environment of Salt Lake region.^[^ ^1^, ^3^ ^]^

**Figure 5:**
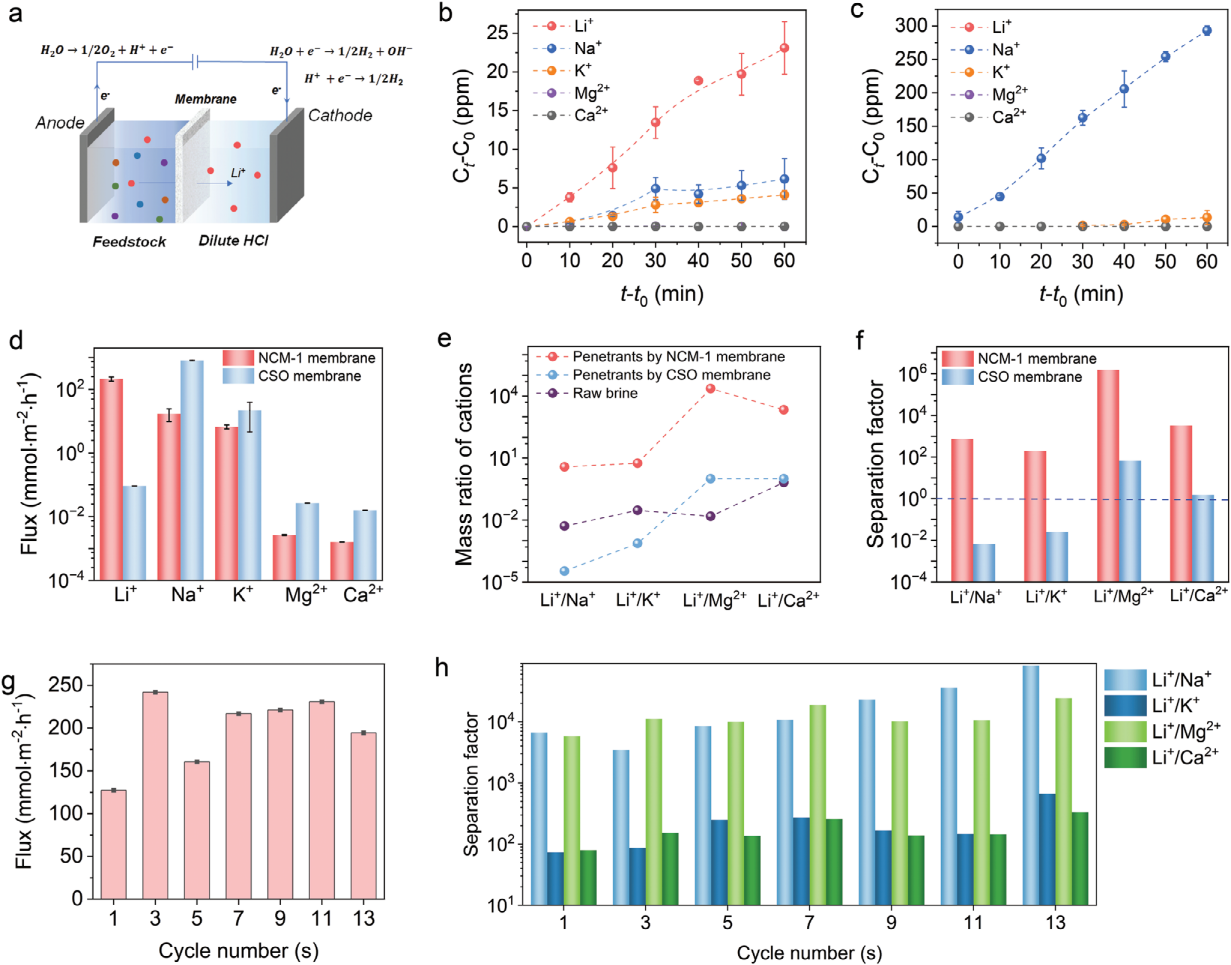
Lithium extraction performance of NCM‐1 and CSO membranes. a) Schematic illustration of lithium extraction equipment. b) Variation of cations concentration in permeate solution by using NCM‐1 membrane. c) Variation of cations concentration in permeate solution by using CSO membrane. d) Fluxes of cations by using brine as the feed solution. e) Mass ratio of cations in raw brine, penetrants in CSO membrane and NCM‐1 membrane system. f) Separation factors (SF) of NCM‐1 membrane and CSO membrane to this raw brine. g) Li^+^ flux in different cycle numbers of lithium extraction from brine. h) Separation factors of Li^+^/Na^+^, Li^+^/K^+^, Li^+^/Ca^2+^, and Li^+^/Mg^2+^ in different cycle numbers. Error bars represent the standard deviation of three measurements of a sample.

Herein, lithium extraction experiments were conducted using a low‐grade brine riched in Na^+^, K^+^, Mg^2+^, and Ca^2+^ as the feedstock. As depicted in **Figure** **5**a, the brine was pumped into the feed compartment, while diluted HCl was filled in the permeate compartment. Subsequently, Li^+^ in the brine can be transported through the NCM‐1 membrane to the permeate solution under an applied electrical driving force. To reduce the influence of Li^+^ in the NCM‐1 membrane on the Li^+^ concentration of the permeate solution, we recorded the ion concentration in the permeate solution after 10 min of operation as C_0_. Figure 5b,c illustrates the variations of cation concentration in the permeate solution as a function of the extracting time. Surprisingly, although the feedstock contains K^+^ and Na^+^ with concentrations dozens or even hundreds of times higher than that of Li^+^, the Li^+^ concentration in the permeate solution with NCM‐1 membrane is much higher than the rival *M* *^n^* ^+^ (Na^+^, K^+^, Mg^2+^, and Ca^2+^) cations. Consequently, the Li^+^ flux surpasses that of the rival cations, indicating the remarkable Li^+^ selective transport of the NCM‐1 membrane even in the existence of extremely high levels of competing cations (as illustrated in Figure 5d). In contrast, the concentration of monovalent cations in the permeate when using a commercial cation exchange membrane CSO membrane follows the order: Na^+^>K^+^>Li^+^, and this concentration trend in the permeate is well in accordance with that in the pristine brine, demonstrating the inability to extract lithium from brines that are rich in Na^+^ or K^+^.

The mass ratio of ions is a crucial parameter for evaluating the quality of the brine.^[^ ^25^, ^79^ ^]^ As illustrated in Figure 5e, both the mass ratio of Li^+^/Mg^2+^ and Li^+^/Ca^2+^ in the permeate solution are significantly improved by using the CSO and NCM‐1 membranes, and the NCM‐1 membrane affords an improvement of 4–6 orders of magnitude, but the CSO membrane delivers only an improvement of two orders of magnitude for Li^+^/Mg^2+^. Notably, the mass ratios of Li^+^/Na^+^ and Li^+^/K^+^ in the penetrants can be individually increased from 0.005 and 0.03 to 3.8 and 5.6 after separation by the NCM‐1 membrane, nearly increasing for two orders of magnitude. Conversely, the mass ratio of Li^+^/Na^+^ and Li^+^/K^+^ in the penetrants even declined by almost two orders of magnitude after separation by the CSO membrane, further confirming that the CSO membrane is incapable for extract Li^+^ from Na^+^ or K^+^‐rich brine.

The separation factor (SF) is typically adopted to assess the separation efficiency of membranes.^[^ ^80^, ^81^ ^]^ In this investigation, we define the SF of Li^+^/*M* *^n^* ^+^ (where *M* *^n^* ^+^ represents Na^+^, K^+^, Mg^2+^, and Ca^2+^) as the ratio of Li^+^/*M* *^n^* ^+^ in the permeate solution to that in the feed solution. As shown in Figure 5f, the CSO membrane exhibits satisfactory separation for Li^+^ from divalent cations, with SF values of 66.0 and 1.5 for Li^+^/Mg^2+^ and Li^+^/Ca^2+^, respectively. However, the SF values of Li^+^/Na^+^ and Li^+^/K^+^ are below 1. Contrarily, the NCM‐1 membrane exhibits exceptional separation performance, with SF values reaching as high as 732.7, 198.0, 1525116.3, and 3273.8 for Li^+^/Na^+^, Li^+^/K^+^, Li^+^/Mg^2+,^ and Li^+^/Ca^2+^, respectively, which were almost the highest record to our best of knowledge. This implies that Li^+^ can be successfully extracted from raw brine which simultaneously riched in Na^+^, K^+^, Ca^2+^, and Mg^2+^ by solely using the NCM‐1 membrane. This outstanding cation separation performance stems from the unique separation manner and may cause a revolutionary alteration in the lithium extraction technique. Besides, the separation factors also correlated to the thickness of the membrane, as the membrane thickness decreased to 0.6 mm, the Li^+^ flux slightly increased, but the separation factors also declined (as described in Figure S17, Supporting Information). Notably, the thin membrane is easy to break during the operation due to the intrinsic brittle of the inorganic membrane.

To evaluate the cycle performance of the NCM‐1 membrane in lithium extraction performance, lithium extraction from brine was repeated for 13 cycles. The Li^+^ flux ranges in 128–250 mmol m^−2^ h^−1^, and it has slightly increased with the enhancement of cycle number (Figure 5g). The separation factors in Figure 5h indicate that the NCM‐1 membrane shows excellent lithium extraction stability even repeated for 13 cycles. Besides, the separation factors also correlated to the thickness of the membrane, as the membrane thickness decreased to 0.6 mm, the Li^+^ flux slightly increased, but the separation factors also declined (as described in Fig. S17). Notably, the thin membrane is easy to break during the operation due to the intrinsic brittle of the inorganic membrane. We have tested the morphology changes of the NCM‐1 membrane after lithium extraction as shown in Figures S18 and S19 (Supporting Information). The top view indicates that the NCM‐1 membrane exposed to brine shows apparent fissures compared to the bottom view, and the cross‐section also demonstrates that obvious crevices formed in the membrane after 13 repeated cycles. The XRD patterns in Figure S20 (Supporting Information) show that the membrane side exposed to brine presents slight diffraction patterns for NaGe_2_(PO_4_)_3_, while the side unexposed to brine has no apparent changes, indicating that lithium extraction repeating under electric driving results to the Na^+^ insertion in NCM‐1 membrane.

Herein, the permeate solution has such low levels of contaminants that it can be directly utilized as a feedstock for Li_2_CO_3_ production without further purification. By precipitating the permeate solution with Na_2_CO_3_ a white powder can be readily produced. The obtained product matches well with Li_2_CO_3_ (PDF# 80‐1307) with uniformly rod‐like morphology (Figure S21, Supporting Information).

Thanks to the excellent Li^+^ selective transport performance, this novel lithium extraction procedure can directly extract lithium from low‐grade brine without the solar‐evaporation process and muti‐steps of purifying, potentially reducing the operation time from >10 months to <10 h. Moreover, conventional lithium extraction strategies often result in significant water loss and substantial solid waste generation during evaporation and the imperfect separation performance of these conventional membranes necessitates multiple purification steps to enhance the quality of the permeate solution.^[^ ^82^ ^]^ By contrast, our one‐step lithium extraction strategy utilizes only electric current, eliminating the water and chemicals consumption, and no waste produced. The energy consumption of lithium extraction for this strategy is 11.1–19.8 Wh mol^−1^ Li^+^, which is higher than that of reports;^[^ ^9^, ^83^ ^]^ however, hydrogen, oxygen, and chlorine were produced during the lithium extraction process which can compensate for the energy consumption. In brief, this approach represents a sustainable, continuous, and highly efficient method for lithium extraction, marking a significant milestone in this field.

## 3 Conclusion

In summary, inspired by the biological ion channel, we fabricated a membrane based on nonporous crystalline materials with a confined and rigid ion channel with the size between the naked Li^+^ and naked Na^+^. Based on the size‐sieving effect, this membrane shows impressive Li^+^/Na^+^ and Li^+^/K^+^ separation with selectivities up to 2707.4 and 5109.8, respectively, surpassing the state‐of‐the‐art membranes in this field for several orders of magnitude. Additionally, combined with the high dehydration‐free energy and the strong electrostatic interaction of the Mg^2+^ and Ca^2+^ with the ion channel, this membrane also exhibits outstanding Li^+^/Mg^2+^ and Li^+^/Ca^2+^ selectivities up to 36161.7, and 60269.6, respectively, which not only outperform the most advanced membranes in this field and it also breakthrough the permeability‐selectivity trade‐off that had restricted this field for decades. By leveraging this performance, we demonstrate the use of this bioinspired membrane in an efficient, one‐step strategy for lithium extraction from low‐grade raw brine simultaneously riched in Na^+^, K^+^, and Mg^2+^, independent time‐consuming, energy‐intensive as well as environmentally unfriendly preprocessing, potentially reducing the operation time from >10 months to <10 h. The separation factor of Li^+^/Na^+^, Li^+^/K^+^, Li^+^/Mg^2+^, and Li^+^/Ca^2+^ in low‐grade brine can reach 732.7, 198.0, 1525116.3, and 3273.8, respectively, which were almost the highest record to the best of our knowledge. This work proposed a novel membrane based on nonporous crystalline materials with sub‐nanoconfined and rigid ion transport channels, and its naked ions sieving behavior will bring a perspective for ion separation, biomimetic engineering, and membrane fabrication.

## Conflict of Interest

The authors declare no conflict of interest.

## Supporting information

Supporting Information

## Data Availability

The data that support the findings of this study are available from the corresponding author upon reasonable request.
