# Supplementary material for: A Bioinspired Membrane with Ultrahigh Li+/Na+ and Li+/K+ Separations Enables Direct Lithium Extraction from Brine
Source: Adv Sci (Weinh). 2024 Jul 19;11(35):2402898. doi: 10.1002/advs.202402898 (PMC11425256; doi:10.1002/advs.202402898)
Supplement: Supplementary file 1 — Supporting Information [file ADVS-11-2402898-s001.docx]

**Supporting Information**

A Bioinspired Membrane with Ultrahigh Li^+^/Na^+^ and Li^+^/K^+^ Separations Enables Direct Lithium Extraction from Brine

Faying Fan ^# 1, 2, 3^, Yongwen Ren ^#^ ^1, 2, 3^, Shu Zhang ^# 1, 2, 3^, Zhilei Tang ^1, 2, 3^, Jia Wang ^1, 2, 3^, Xiaolei Han ^1, 2, 3^, Yuanyuan Yang ^1, 2, 3^, Guoli Lu ^1, 2, 3^, Yaojian Zhang ^1, 2, 3^, Lin Chen ^1, 2, 3^, Zhe Wang ^1, 2, 3^, Kewei Zhang ^5^, Jun Gao, ^1, 2, 3^ Jingwen Zhao * ^1, 2, 3^, Guanglei Cui * ^1, 2, 3^, Bo Tang * ^4^

1. Qingdao Industrial Energy Storage Research Institute, Qingdao Institute of Bioenergy and Bioprocess Technology, Chinese Academy of Sciences, Qingdao 266101, China.

2. Shandong Energy Institute, Qingdao 266101, China.

3. Qingdao New Energy Shandong Laboratory, Qingdao 266101, China

4. Laoshan Laboratory

5. Qingdao University

Email: (zhaojw@qibebt.ac.cn; cuigl@qibebt.ac.cn; tangb@sdnu.edu.cn)

**1. Experimental**

**1.1 Chemicals and reagents**

Li_1.5_Al_0.5_Ge_1.5_(PO_4_)_3_, Li_1.5_Al_0.5_Ti_1.5_(PO_4_)_3_, and Na_2.5_Zr_2_Si_1.5_(PO_4_)_3_ powder were purchased from Kejing technology company. KCl, LiCl, NaCl, CaCl_2_, and MgCl_2_ of analytical grade were obtained from China National Pharmaceutical Group Industry Co., Ltd. (Beijing, China). All reagents and solvents were commercially available and used as received. Commercial monovalent selective [cation exchange membranes](https://www.sciencedirect.com/topics/engineering/cation-exchange-membrane) (CSO membrane, monovalent cation selective membrane, AGC Engineering Co., Ltd.) were received from Beijing Jierui Environment Protection Technology Co., Ltd. Deionized water with conductance of 1.82*10^7^ Ω was used throughout the experiments.

**1.2 Preparation and characterization of ceramic membrane**

The bioinspired Li_1.5_Al_0.5_Ge_1.5_(PO_4_)_3_ (NCM-1), Li_1.5_Al_0.5_Ti_1.5_(PO_4_)_3_ (NCM-2), and Na_2.5_Zr_2_Si_1.5_(PO_4_)_3_ (NCM-3) membranes were obtained by a modified method ^1,2^. Typically, the acquired Li_1.5_Al_0.5_Ge_1.5_(PO_4_), Li_1.5_Al_0.5_Ti_1.5_(PO_4_), and Na_2.5_Zr_2_Si_1.5_(PO_4_)_3_ powders were individually cold isostatically pressed into 20 mm diameter discs under 50 MPa and subsequently annealed at different temperatures for 12 hours, maintaining a consistent thickness of 1 mm. For the NCM-1 and NCM-2 membranes, the maximum annealing temperature was set at 870 °C, whereas the maximum annealing temperature was 1150 °C for the NCM-3 membrane. The crystal phase of the obtained ceramic membrane was characterized by XRD using a Bruker diffractometer with Cu Kα radiation source. The morphology and EDX of the membranes were observed using Hitachi S-4800 cold field emission scanning electron microscopy (SEM). The Li-ion diffusion coefficient was measured by the pulsed-field Gradient-NMR measurement, which was carried out on a Bruker AVANCE NEO 500WB using a commercial Bruker Diff50 probe with a maximum gradient of 1700 G·cm^-1^ at 25 °C. The decay of the signal as a function of the gradient strength can be described by Equation S1 where γ stands for the gyromagnetic ratio of 19F and D for the self-diffusion coefficient, and the experimental data was presented in Fig. S4.

$$A_{echo}\left( g^{2} \right)=\sum_{i}A_{i}e^{(-D_{i}\gamma^{2}\delta^{2}\left( \Delta-\frac{\delta}{3} \right)g^{2})}$$

**1.3 Cations transport performance test**

The transportation performance of cations was carried out using homemade equipment as shown in Fig. S5, which contains a feed and permeate compartment, and a pair of titanium plates coated with ruthenium oxide was used as the cathode and anode. The feed and concentrated compartment were separately filled with feedstock and permeate solution, and the membranes were respectively placed between the feed and permeate compartment. 3 V of direct voltage was used as the driving force, and the effective area of the membrane was 3.14 cm^2^. In the Li^+^ flux testing, 0.1 M of LiCl aqueous solution and 0.01 M of HCl solution were used as the feed and permeate solution. For the cations separating selectivity test, a mixed cations aqueous solution with a total concentration of 0.5 M (contains 0.1 M of LiCl, NaCl, KCl, MgCl_2_, and CaCl_2_) was used as the feed solution, after operation for 10 min the concentration in permeate solution recorded as C_0_, the concentration of the cations in the permeate solution at time of *t* recorded as C*_t_*, the concentration of cations was tested using ICP-MS to calculate the flux.

Cations flux through membrane was measured by the following equation ^3^:

$$J_{N^{n+}}=\frac{(C_{t}-C_{0})\cdot V}{A_{m}\cdot\Delta t}$$

Where *C*_0_ and *C*_t_ are the concentrations of the cations in the permeate solution at the beginning (*t*=10 min) and end (*t*=70 min). *V* is the volume of the permeate solution (20×10^-3^ L), *A_m_* is the effective surface area of the membrane (3.14×10^-4^ m^2^), and $\Delta t$ is one hour.

The selectivity was determined using the follows equation ^3,4^:

$$S_{{Li}^{+}/M^{n+}}=\frac{J_{{Li}^{+}}\cdot C_{M^{n+}}}{J_{M^{n+}}\cdot C_{{Li}^{+}}}$$

Where $J_{{Li}^{+}}$and $J_{M^{n+}}$ are the flux of Li^+^ and *M*^n+^, respectively. And the $C_{{Li}^{+}}$ and $C_{M^{n+}}$ are the concentration of Li^+^ and *M*^n+^ in the feed solution, respectively.

**1.4 Calculation method**

All first-principles calculations are performed using the plane-wave projector-augmented wave method, as implemented in the Vienna ab initio simulation package (VASP) ^5,6^ within the MedeA technology platform ^7^. The Perdew−Burke−Ernzerhof (PBE) form of generalized gradient approximation (GGA) is chosen as the exchange−correlation potential ^8^. The energy cutoff of 520 eV was used for structural relaxation. The conjugated gradient method optimizes the geometry with the convergence threshold of 10^-5^ eV in energy and 0.01 eV/Å in force, respectively. NCM-1, NCM-2, and NCM-3 modes were established and optimized to calculate the relevant parameters.

The formation energy of *M* (*M*=Na^+^, K^+^, Mg^2+^, and Ca^2+^) entering into the NCM-1 lattice is calculated by the following equation:

$$E_{f}=E_{NCM-1\_M}+nE_{Li}-E_{NCM-1}-E_{M}$$

Where $E_{NCM-1}$ represents the energy of NCM-1, $E_{NCM-1\_M}$ is the energy of Li^+^ in NCM-1 replaced by *M* ion, $E_{Li}$ is the energy of Li atom in the bulk structure, and $E_{M}$ is the energy of *M* atom in the bulk structure, respectively. And *n*=1 for Na^+^ and K^+^, *n*=2 for Mg^2+^ and Ca^2+^. Li atoms are located in the large voids/channels of the framework, occupying 3 different Wyckoff sites Li(1) (6*b*, with an oxygen environment of 6-fold coordination), Li(2) (18*e*, with an oxygen environment of 8-fold coordination) and Li(3) (36*f*, with an oxygen environment of 5-fold coordination) ^9^, and all of the *M* ions tend to stabilize at the 6*b* site.

The bond valence energy landscape (BVEL) method is used to simulate Li^+^ diffusion pathways, and the iso-surface is displayed by the VESTA visualization package. The bottleneck size is the radius of the inscribed circle of a triangle composed of nearby oxygen atoms around the narrowest migration channel. The *M* ion migration path and energy barrier (*M*=Li^+^, Na^+^, K^+^, Mg^2+^, and Ca^2+^) are calculated using the DFT-based climbing image nudged elastic band (CI-NEB) method.

**1.5 Lithium extraction from brine**

Yili Ping Salt Lake brine was applied to test the lithium extracting performance, and the chemical compositions of the brine are listed as follows:

**Table S1** The chemical composition of the brine ^10^.

|  | Li^+^ | Na^+^ | K^+^ | Ca^2+^ | Mg^2+^ |
| --- | --- | --- | --- | --- | --- |
| Simulated brine (g/L) | 0.25 | 48.39 | 8.41 | 0.38 | 16.37 |

The feed and permeate compartment were separately filled with brine and 0.01 M of HCl. The cations concentration in the permeate solution was detected using ICP-MS. The separation factor (SF) refers to the mass ratio change of Li^+^ to the relative co-existed cations *M* in the feed and permeate solution^11-13^. It is defined as:

$$SF=\frac{C_{Li(P)}/C_{M(P)}}{C_{Li(f)}/C_{M(f)}}$$

Where, *C_M(f)_* and *C_Li(f)_* refer to the concentration of *M*^+^ and Li^+^ in the brine, respectively. *C_M(P)_* and *C_Li(p)_* refer to the concentration of cations in the permeate solution at a certain time.

**1.6 Li_2_CO_3_ production**

The permeate solution was used as the feedstock for Li_2_CO_3_ production. Typically, 2 M Na_2_CO_3_ aqueous solution was dropwise added into the permeate solution and stirred at 90 ^o^C for 2 h, and white precipitation was obtained after distilling the solution by evaporating. The precipitation was washed with hot water three times and filtrated. The crystal structure and morphology of the obtained Li_2_CO_3_ were observed using XRD and SEM.


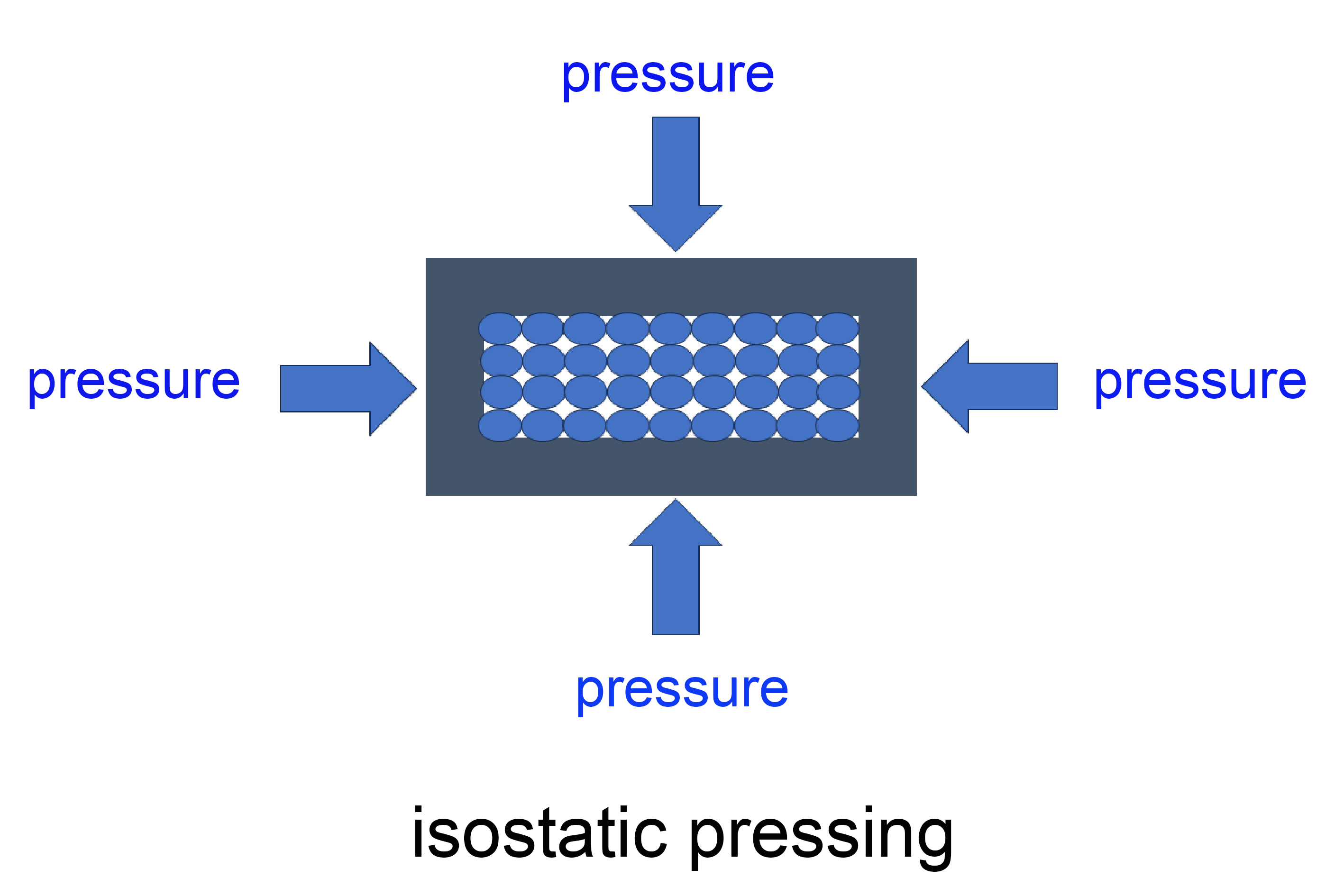


**Schematic diagram 1** Description of isostatic pressing.


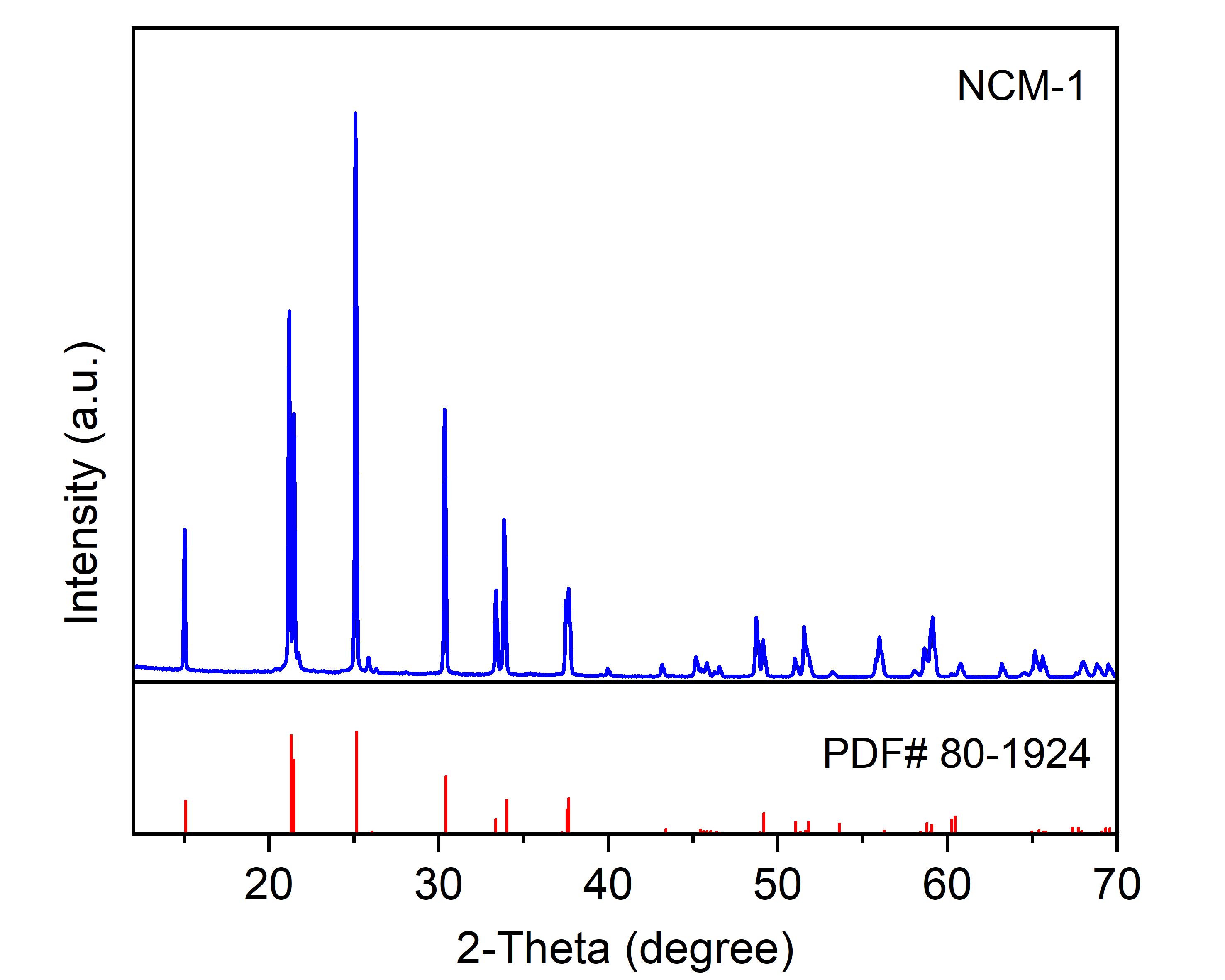


**Fig. S1** XRD pattern of the NCM-1 membrane.


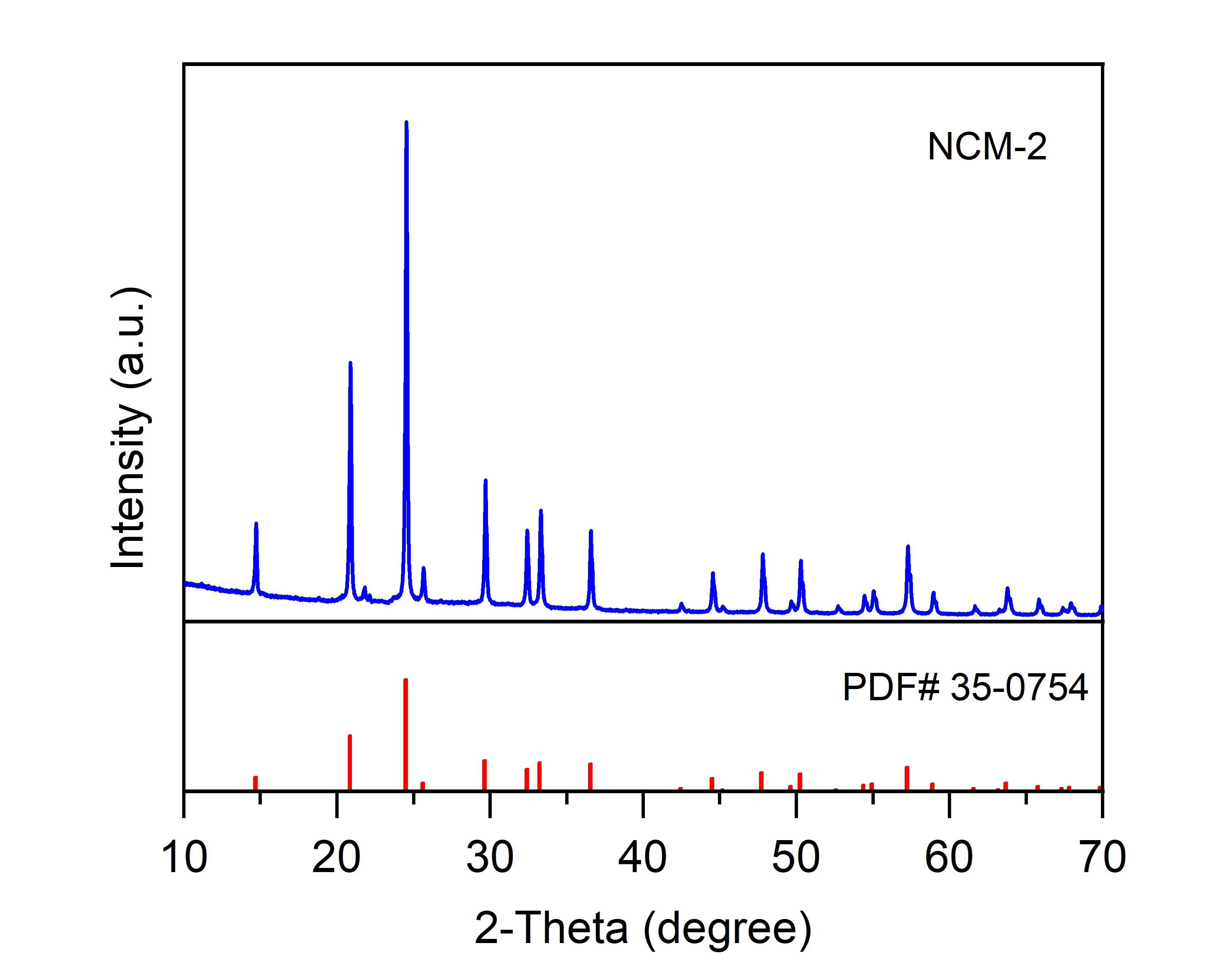


**Fig. S2** XRD pattern of the NCM-2 membrane.


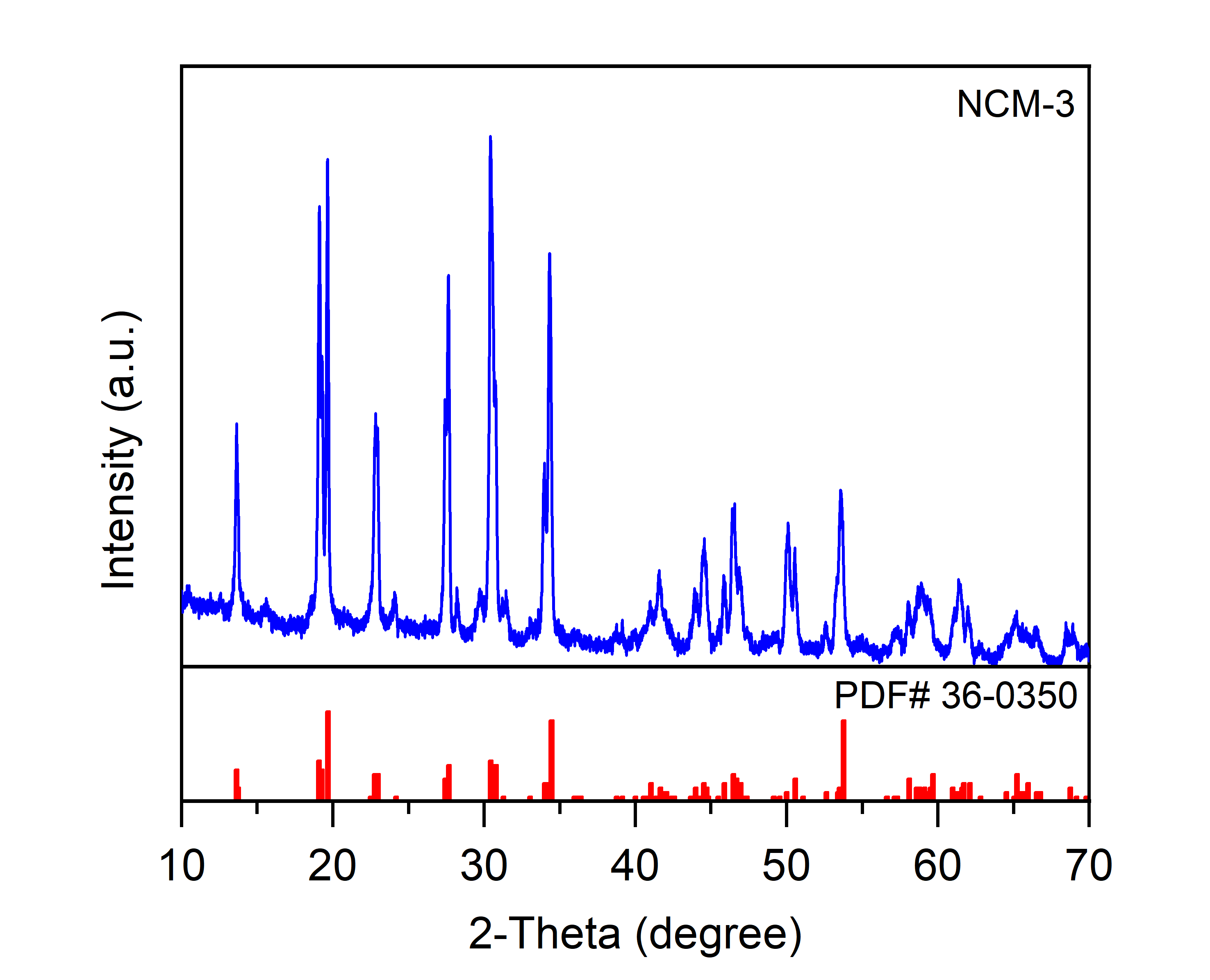


**Fig. S3** XRD pattern of the NCM-3 membrane.

**
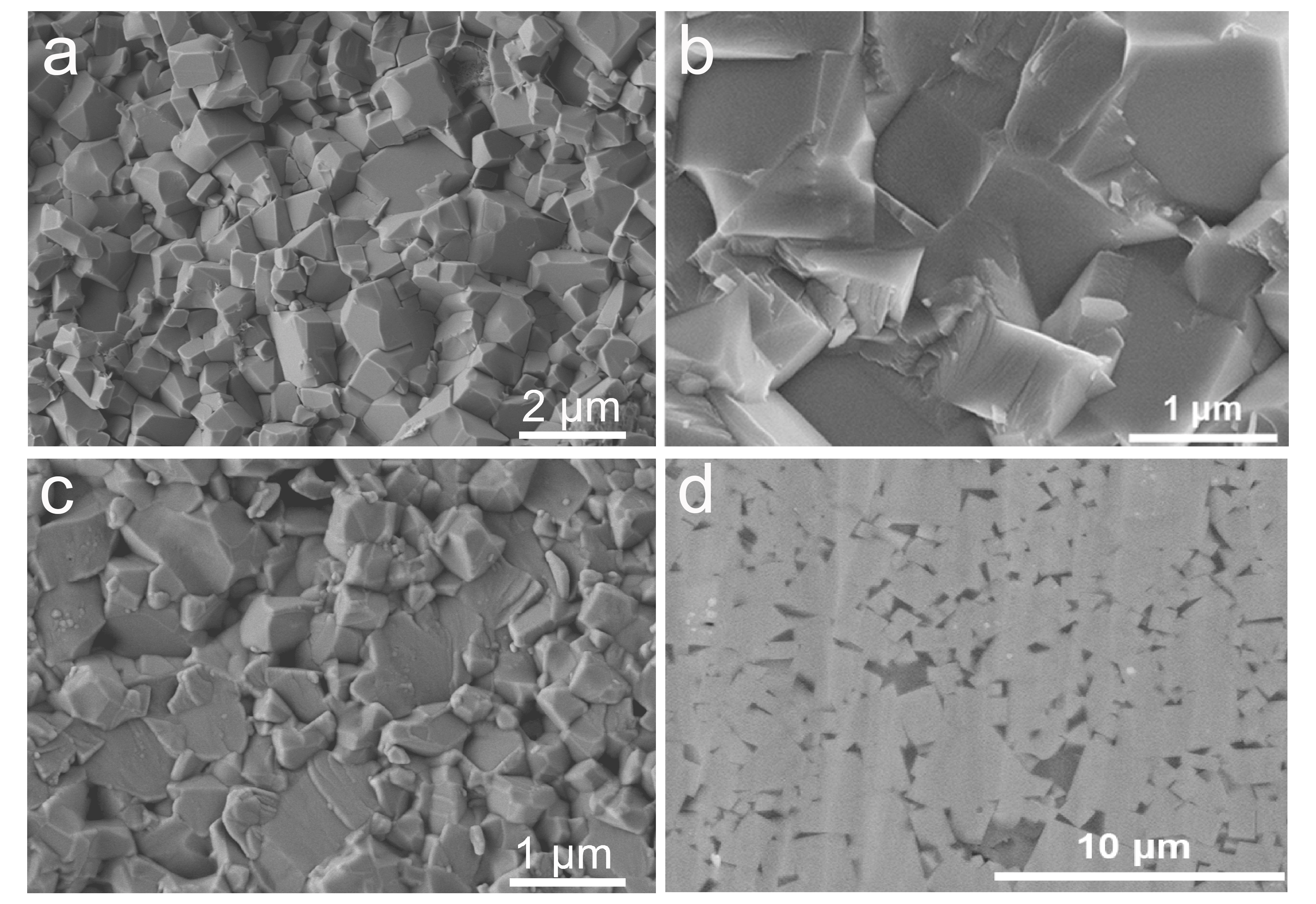
**

**Fig. S4** Cross-sectional SEM images of the NCM-1 membrane (a), NCM-2 membrane (b), NCM-3 membrane (C), and the ion-beam polished cross-sectional SEM images of the NCM-1 membrane.


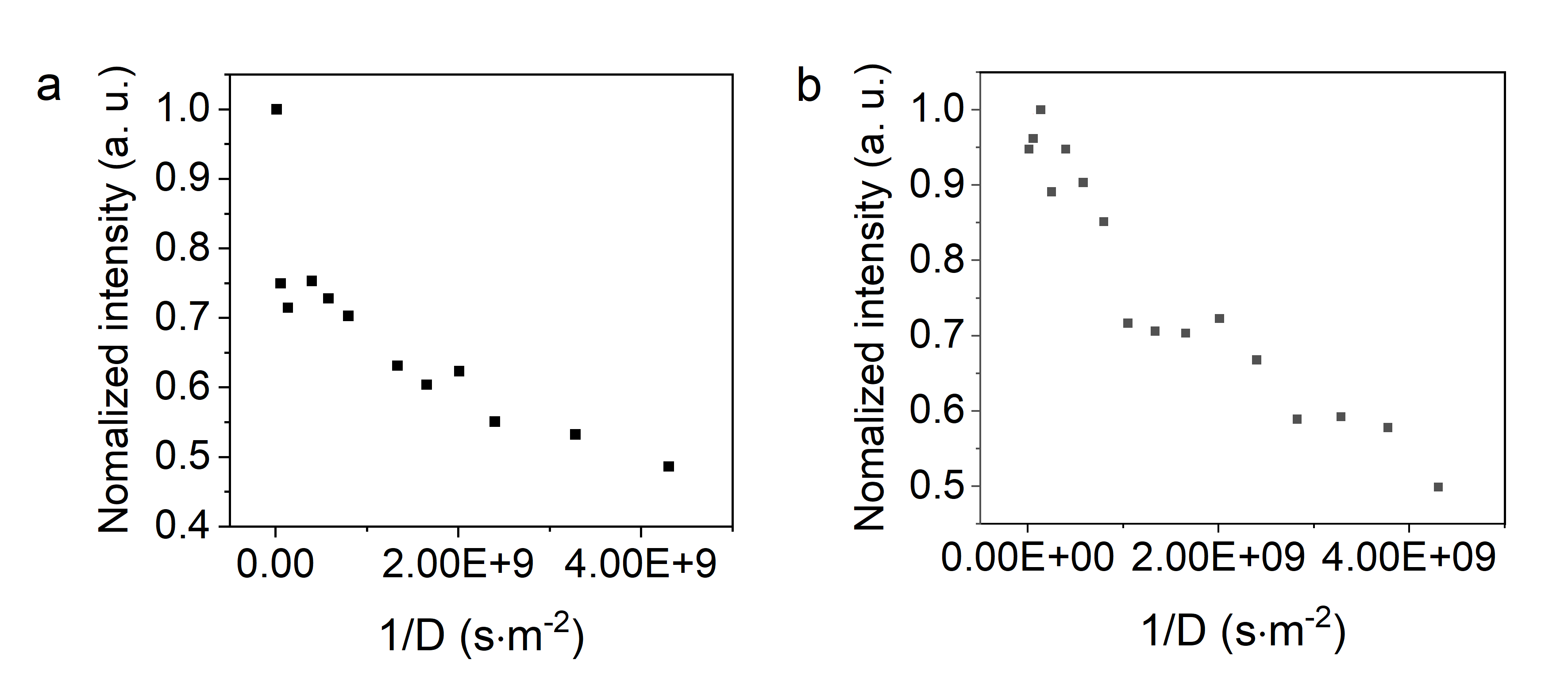


**Fig. S5** Stimulated echo intensity as a function of the squared gradient amplitude from a 7Li pulsed field gradient NMR experiment. (a) NCM-1 powder; (b) NCM-1 membrane.


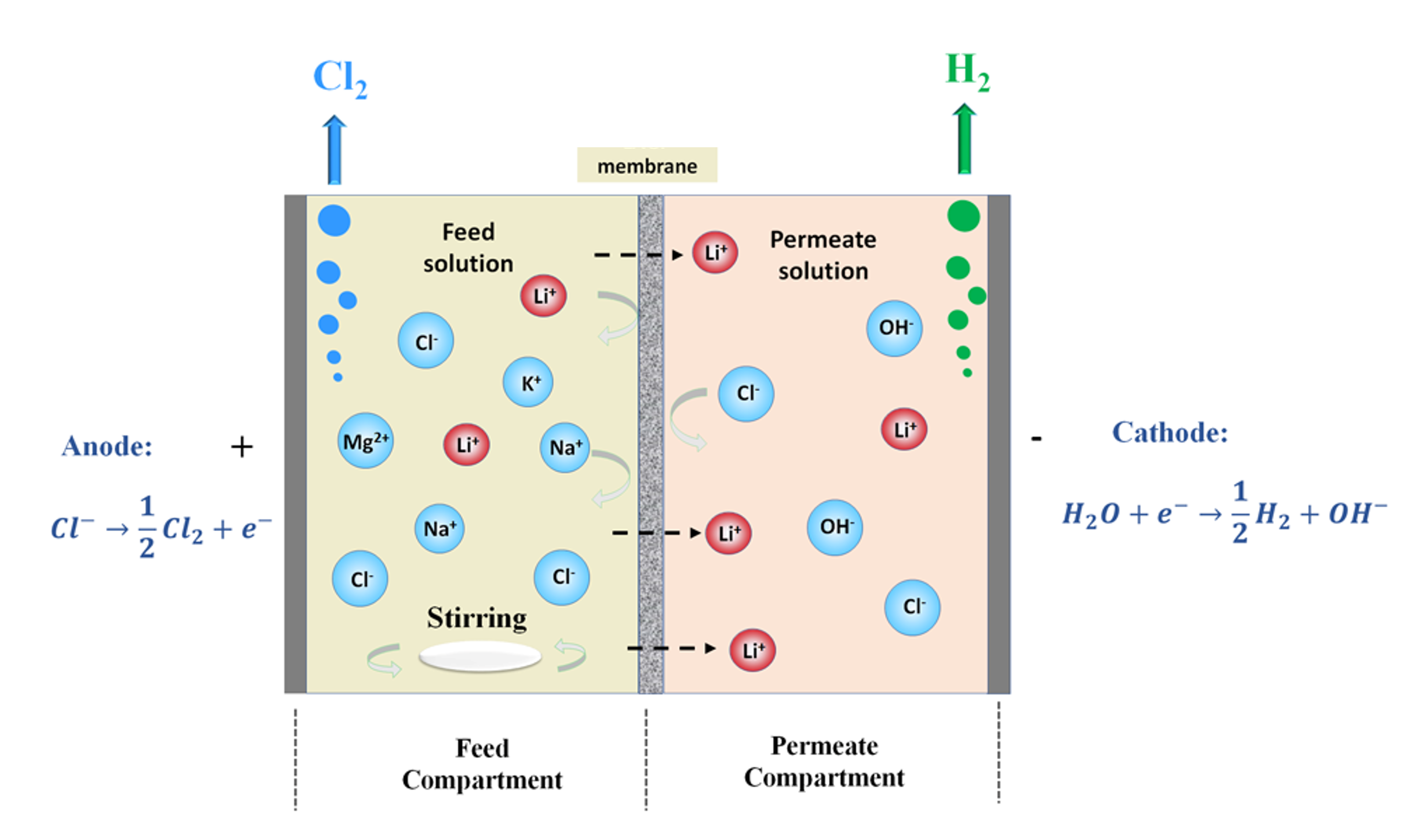


**Fig. S6** The apparatus for testing of cation permeate performance.


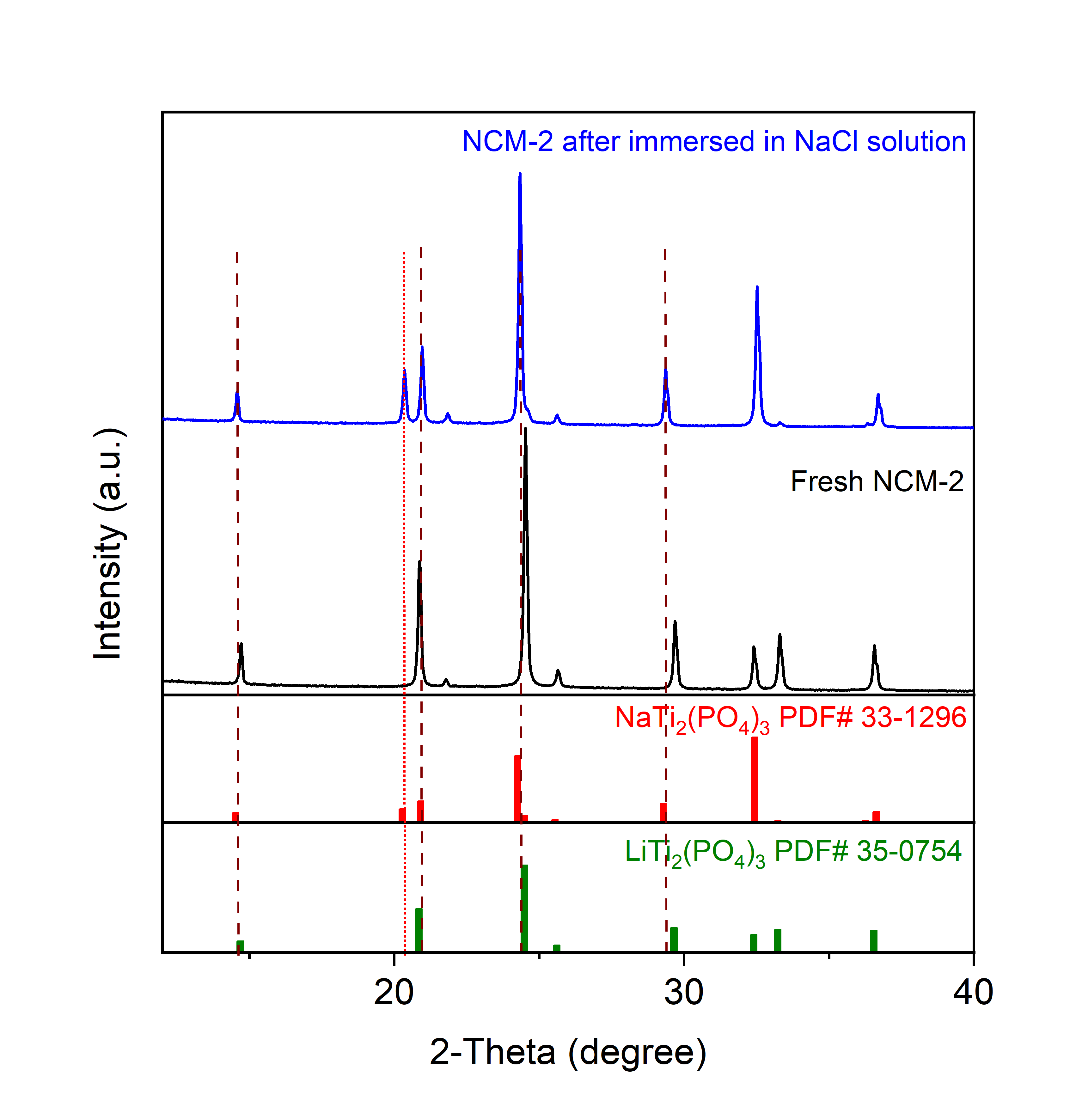


**Fig. S7** XRD patterns of the fresh NCM-2 membrane and the membrane after being immersed in 0.1 M NaCl solution for 7 days.

**
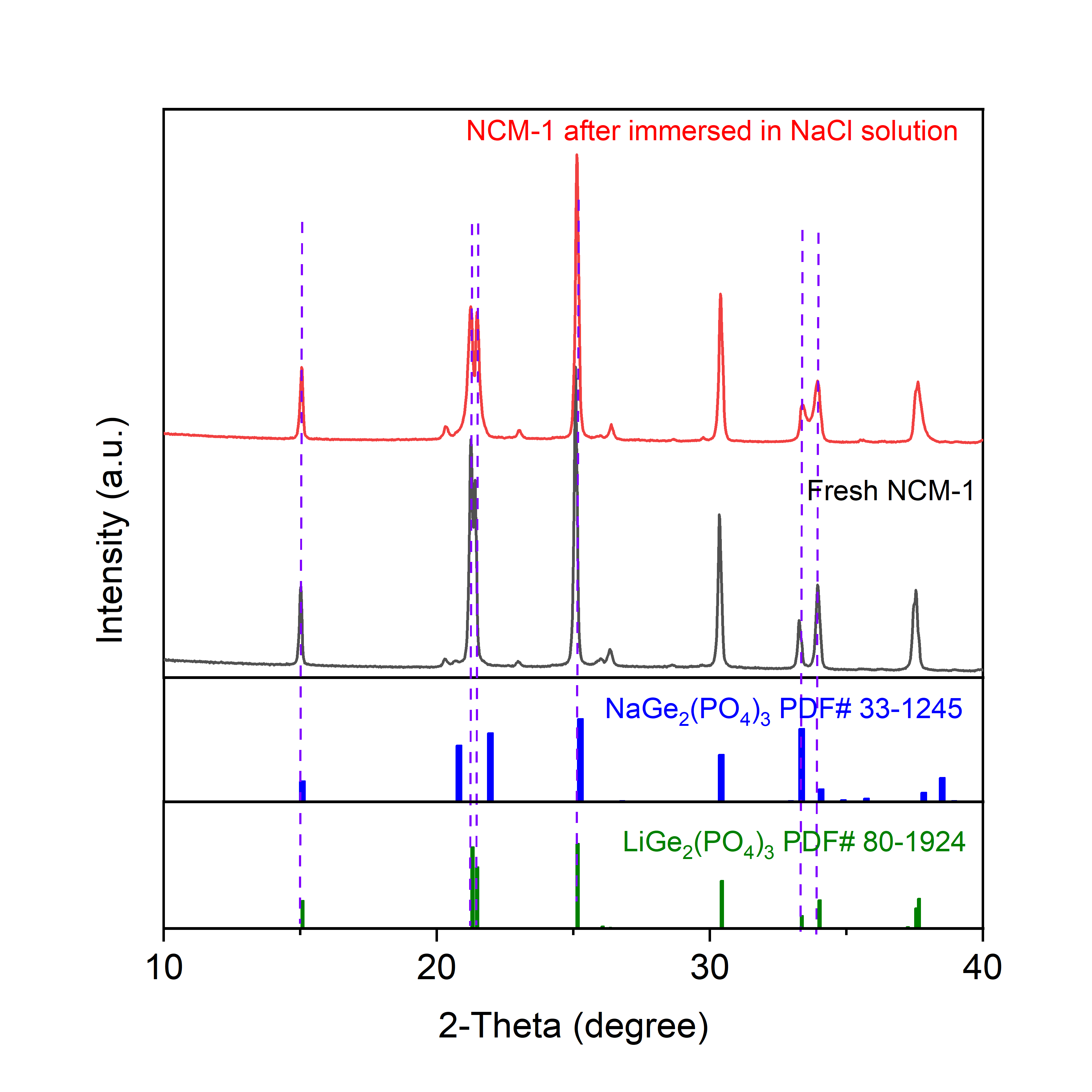
**

**Fig. S8** XRD patterns of the fresh NCM-1 membrane and the membrane after being immersed in 0.1 M NaCl solution for 7 days.

Table S2 The substitution energy and lattice parameters of substitution Li^+^ in NMC-2 membrane with Na^+^.

| Sample | Substitution energy | a | b | c | volume |
| --- | --- | --- | --- | --- | --- |
| NCM-2 |  | 8.59502 | 8.6115 | 21.12022 | 1356.4979 |
| NCM-2_1Na | -0.181 | 8.6100 | 8.5951 | 21.3364 | 1370.1704 |
| NCM-2_2Na | -0.183 | 8.5994 | 8.5851 | 21.5025 | 1377.5664 |
| NCM-2_3Na | -0.081 | 8.5875 | 8.5852 | 21.6679 | 1384.1202 |
| NCM-2_4Na | -0.100 | 8.5847 | 8.5797 | 21.8049 | 1391.3138 |
| NCM-2_5Na | 0.239 | 8.5782 | 8.5688 | 21.9445 | 1396.4380 |
| NCM-2_6Na | 0.656 | 8.5982 | 8.5962 | 21.9252 | 1402.8815 |
| NCM-2_7Na | 0.956 | 8.6125 | 8.5976 | 21.9189 | 1406.1612 |
| NCM-2_8Na | 0.953 | 8.6129 | 8.5958 | 21.9659 | 1408.8041 |
| NCM-2_9Na | 0.995 | 8.6182 | 8.6177 | 21.9402 | 1411.0336 |
| NCM-1 |  | 8.3841 | 8.39013 | 20.8701 | 1272.4199 |
| NCM-1_1Na | 0.135 | 8.3619 | 8.35988 | 21.0528 | 1276.4427 |
| NCM-3 |  | 9.1021 | 9.1021 | 22.8354 | 1638.4144 |
| NCM-3_1Li | 0.116 | 9.0908 | 9.0908 | 22.9139 | 1639.9693 |

Note: NCM-2_1Na is one Na^+^ substitute one Li^+^ in NCM-2; NCM-2_2 Na is two Na^+^ substitute two Li^+^ in NCM-2; similar NCM-2_*x*Na is the number of Na^+^ substitute Li^+^ in NCM-2 is *x*.

**Table S3** Comparison of Li^+^ flux and separation selectivity (*S*) of membranes under electrical potential driven.

|  | *Materials* | *Experimental condition (mol· L^-1^)* | *Current density (mA·cm^-2^)* | *S (Li^+^/Na^+^)* | *S*  *(Li^+^/K^+^)* | *S (Li^+^/Mg^2+^)* | *S*  *(Li^+^/Ca^2+^)* | *Flux*  *(mmol·m^-2^· h^-1^)* | *Ref.* |
| --- | --- | --- | --- | --- | --- | --- | --- | --- | --- |
| 1 | POC-CC3 | 0.1 | 5 | 0.4 | 0.28 | 248 |  | ~500 | ^3^ |
| 2 | UiO-66-SO_3_H | 0.1 | 5 | 0.31 | 0.15 | 776 |  | ~360 | ^14^ |
| 3 | PVA-based membranes | 0.1 | 2.12 |  |  | 5.2 |  | ~180 | ^15^ |
| 4 | ZSM-5 | 0.1 | 30 |  |  | 3.7 |  | ~1200 | ^4^ |
| 5 | Ti_3_C_2_T_x_/PSS | 0.2 |  | 15.5 | 12.7 | 28 |  | ~80 | ^16^ |
| 6 | COF | 0.1 |  | 0.32 | 0.28 | 217 |  | ~60 | ^17^ |
| 7 | MLM-EDTA | 0.2 |  | 0.5 | 0.2 | ~28 |  | ~20 | ^18^ |
| 8 | PSS/HKUST-1 | 0.5 |  | 35 | 67 | 1815 |  | ~6750 | ^19^ |
| 9 | UiO-66-NH_2_ | 0.1 | 5 | 2 | 0.3 | 60 |  | ~70 | ^20^ |
| 10 | UiO-66(Zr/Ti)-NH_2_ | 0.1 | 10 | 0.85 |  | 11.38 |  | 1950 | ^21^ |
| 11 | P-COOH-QSQ membrane | 0.1 | 2.12 |  |  | 5.16 |  | ~120 | ^15^ |
| 12 | QAIPA-20 membrane | 0.1 | 2.12 | 0.19 | 0.32 | 8 |  | ~13 | ^22^ |
| 13 | Janus-charged M-CEMs | 0.1 | 5 | 0.1 |  | 14.11 |  | ~1800 | ^23^ |
| 14 | 12-crown-4-functionalized membrane | 0.144 | 0 | ~2.3 |  |  |  | ~720 | ^24^ |
| 15 | Bioinspired NCM-1 membrane | 0.1 M single-salt system | ~0.5 | 2707.4 | 5109.8 | 336161.7 | 60269.6 | 575.8 | This work |
| 16 | Bioinspired NCM-1 membrane | 0.1 M mixed-salt system | ~0.5 | 1325.2 | 13695.9 | 25283.7 | 14048.2 | 234.8 | This work |


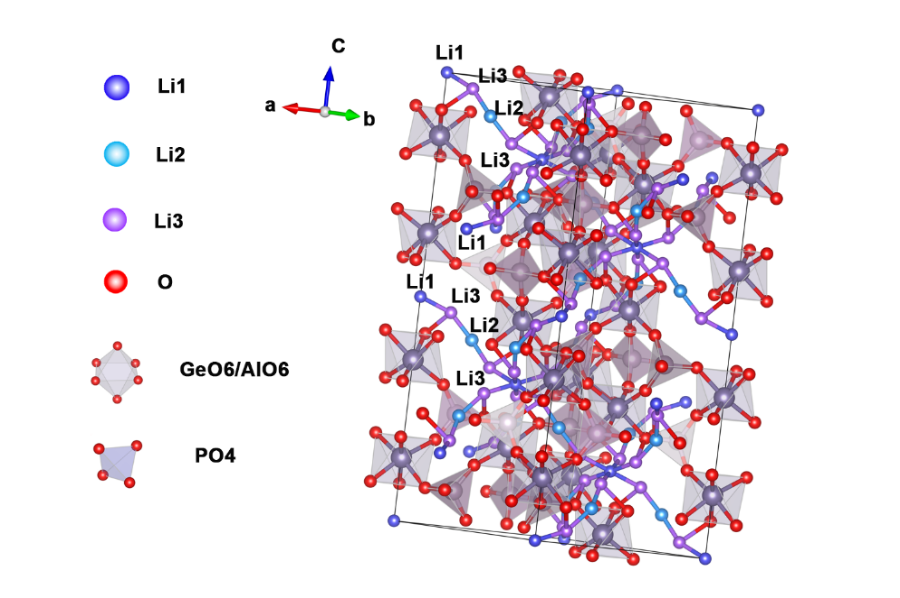


**Fig. S9** The Li atoms in the NCM-1. Li atoms are located in the large voids/channels of the framework, occupying 3 different Wyckoff sites Li(1) (6*b*, with an oxygen environment of 6-fold coordination), Li(2) (18*e*, with an oxygen environment of 8-fold coordination) and Li(3) (36*f*, with an oxygen environment of 5-fold coordination).


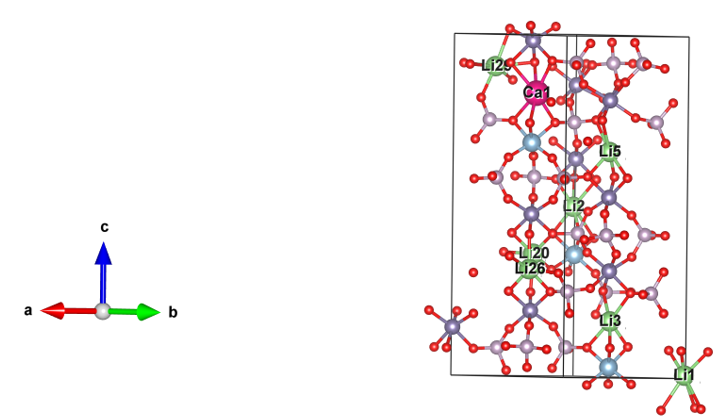


**Fig. S10** The crystal structure of Ca^2+^ substituted NCM-1, and the Ca^2+^ are located in 6*b*.


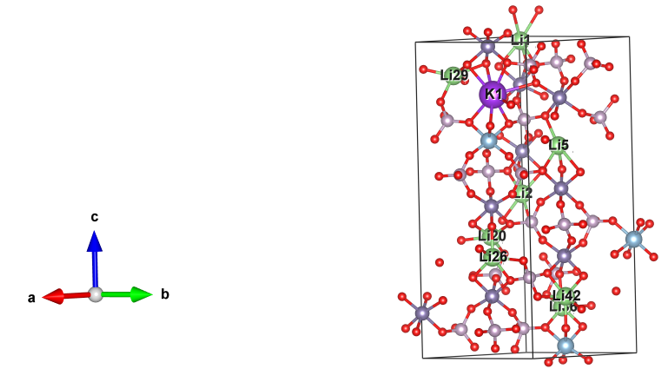


**Fig. S11** The crystal structure of K^+^ substituted NCM-1, and the K^+^ are located in 6*b*.


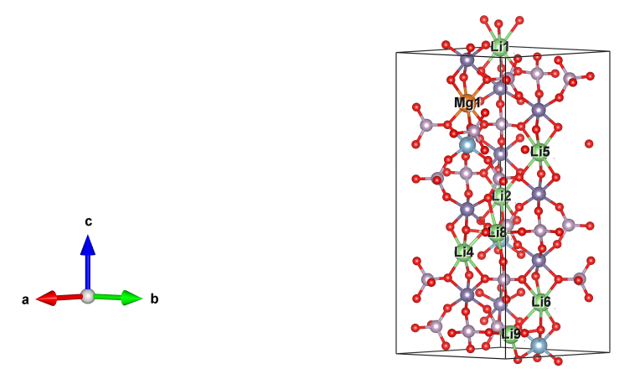


**Fig. S12** The crystal structure of Mg^2+^ substituted NCM-1, and the Mg^2+^ are located in 6*b*.


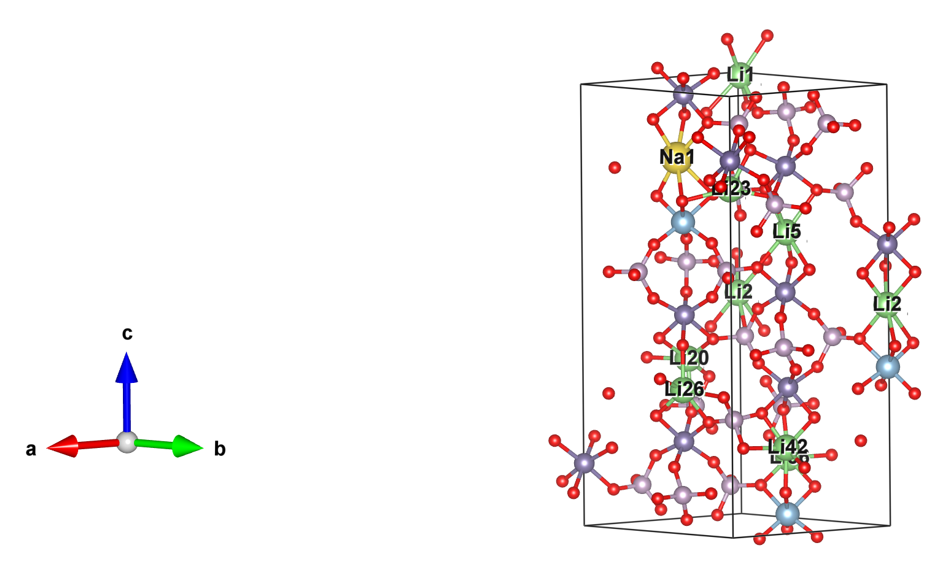


**Fig. S13** The crystal structure of Na^+^ substituted NCM-1, and the Na^+^ are located in 6*b*.

**
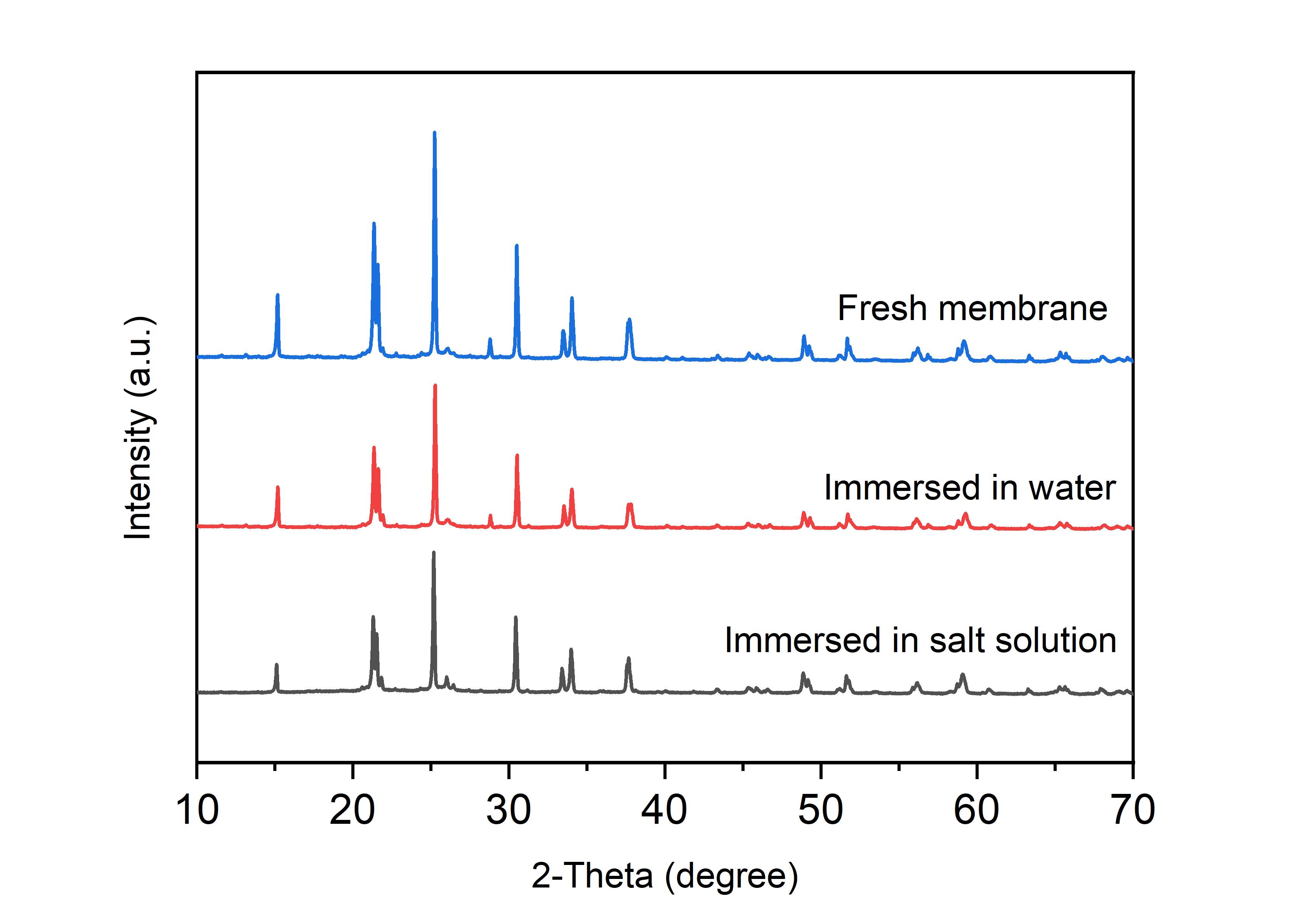
**

**Fig. S14** XRD patterns for fresh NCM-1 membrane, and NCM-1 membranes after immersed in H_2_O and 0.1 M mixed salt solution for 7 days.


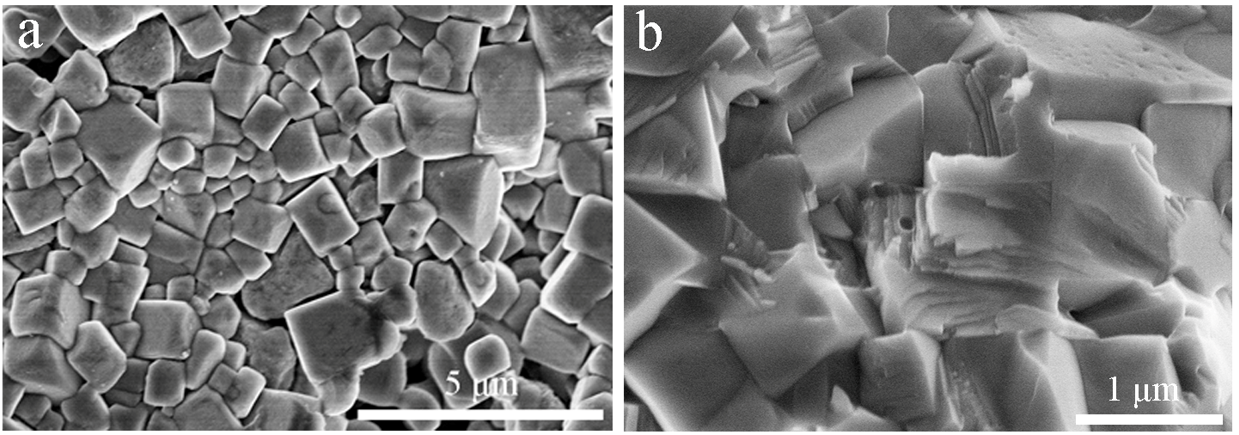


**Fig. S15** SEM images of (a) top-view and (b) crossing-section of the NCM-1 membrane after 3 cycles.


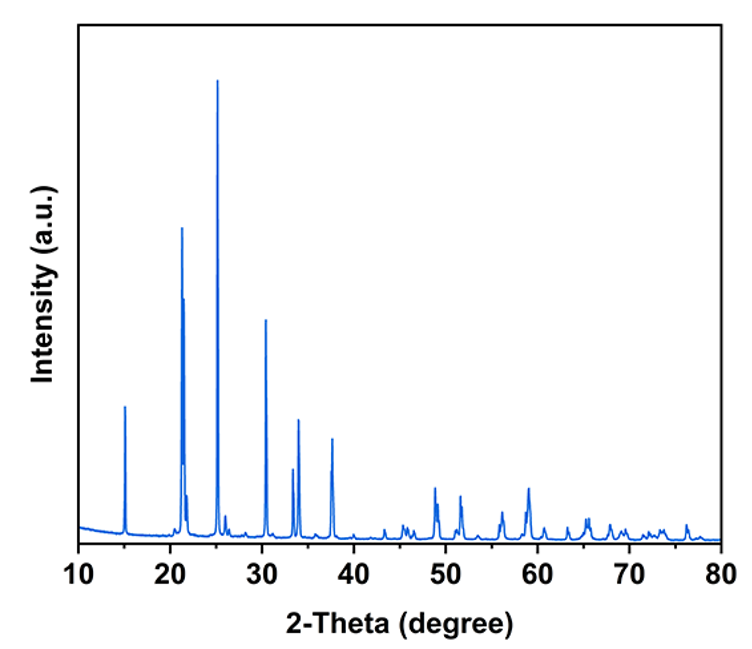


**Fig. S16** XRD pattern for NCM-1 membrane after operating in 0.1 M of mixed salt solution.

**
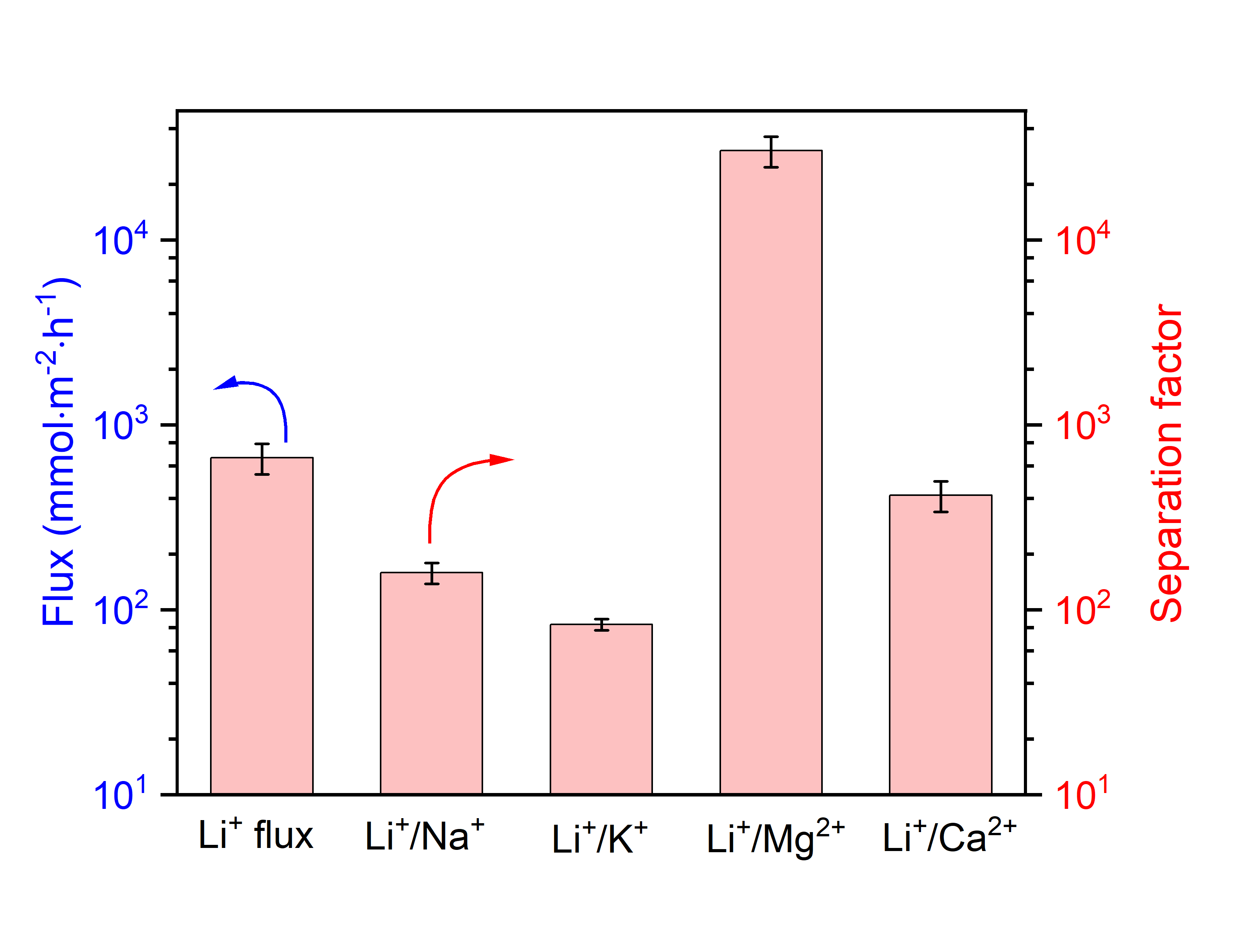
**

**Fig. S17** Li^+^ flux and separation factors of the NCM-1 membrane with a thickness of 0.6 mm.


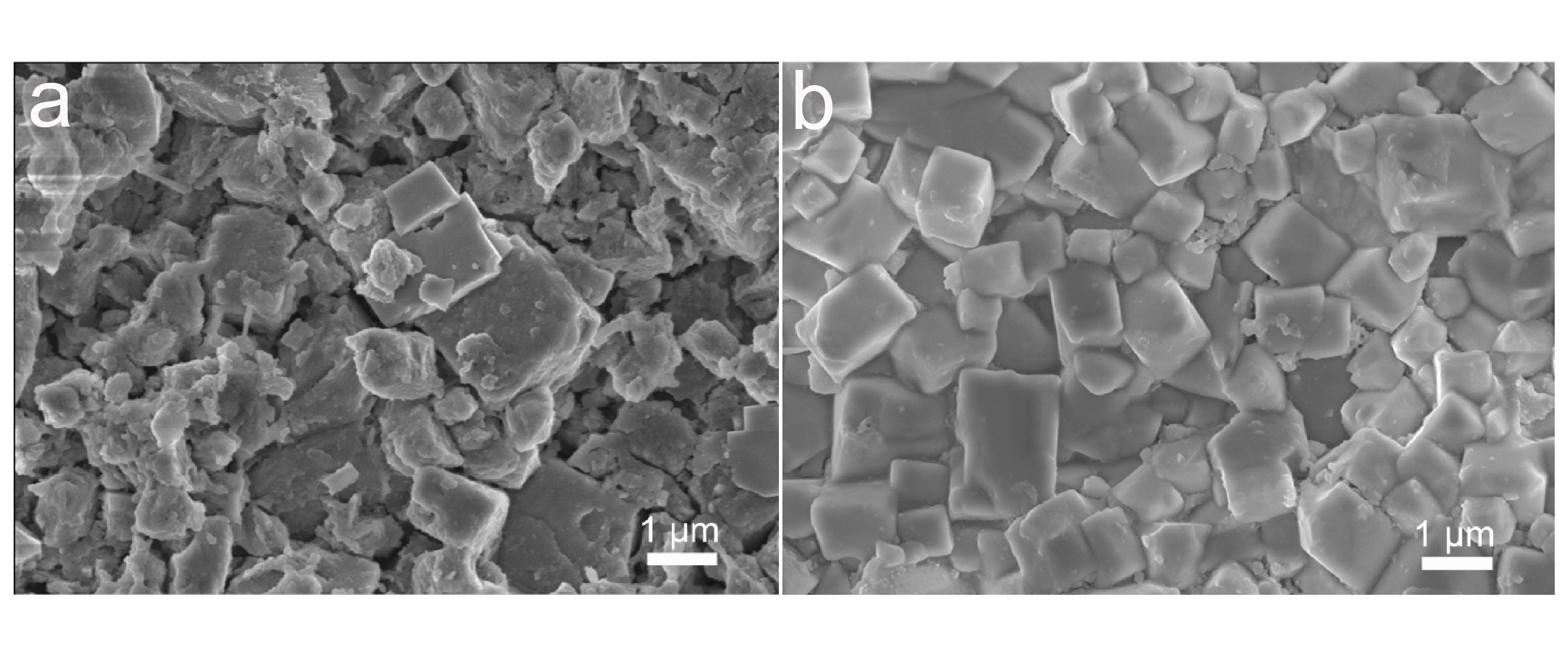


**Fig. S18** SEM images of (a) top-view exposed to the brine and (b) bottom-view of the NCM-1 membrane after repeated for 13 cycles.

**
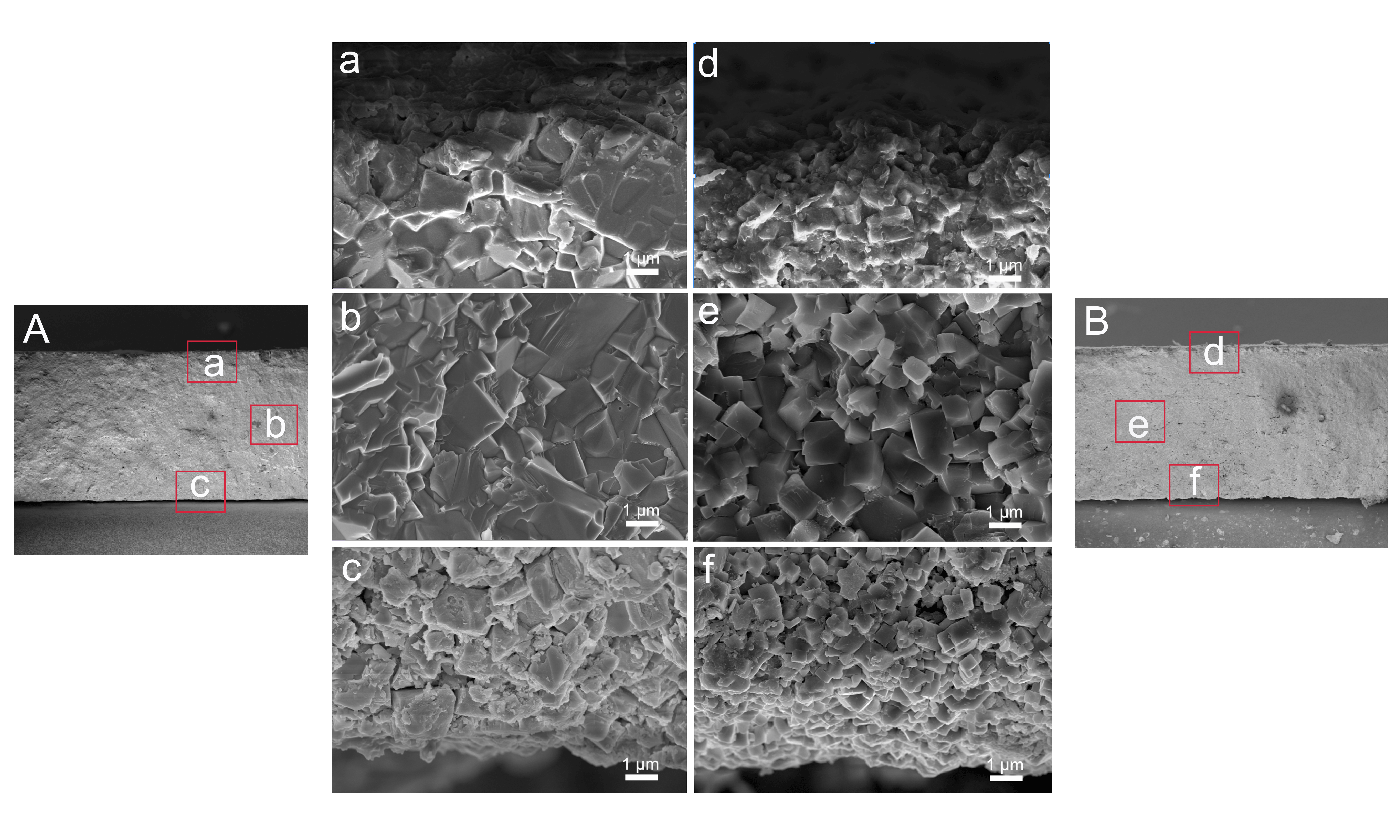
**

**Fig. S19** SEM images of the crossing-section of the NCM-1 membrane: (A) the whole section of NCM-1 membrane after 3 cycles; (a, b, and c) The top, middle, and bottom section of the membrane after 3 cycles; (B) the whole section of NCM-1 membrane after 13 cycles; (d, e and f) The top, middle, and bottom section of the membrane after 13 cycles. The bottom sections are exposed to brine.

**
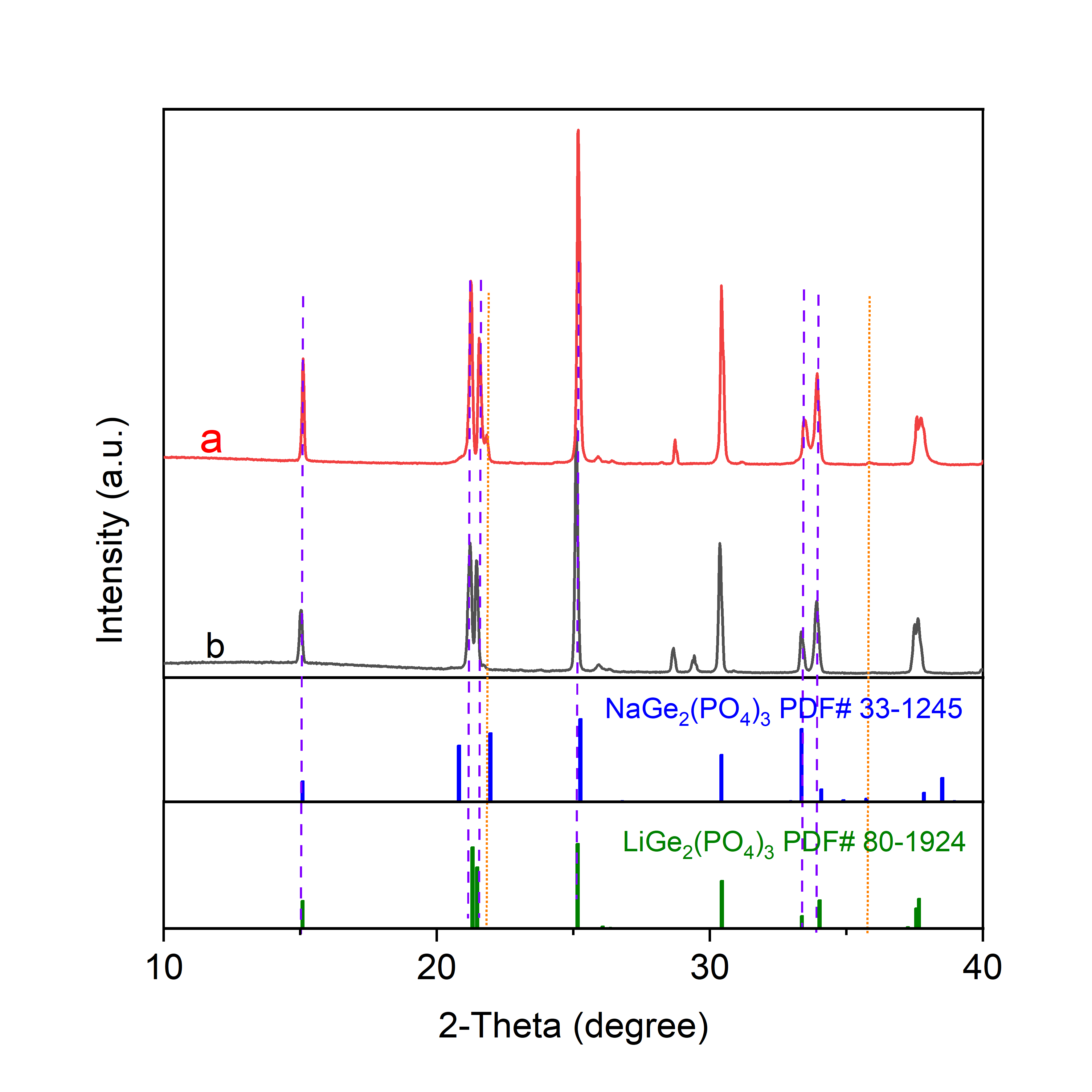
**

**Fig. S20** XRD patterns of NCM-1 membrane after repeated lithium extraction performance for 13 cycles. (a) the side exposed to brine; (b) the side unexposed to brine.


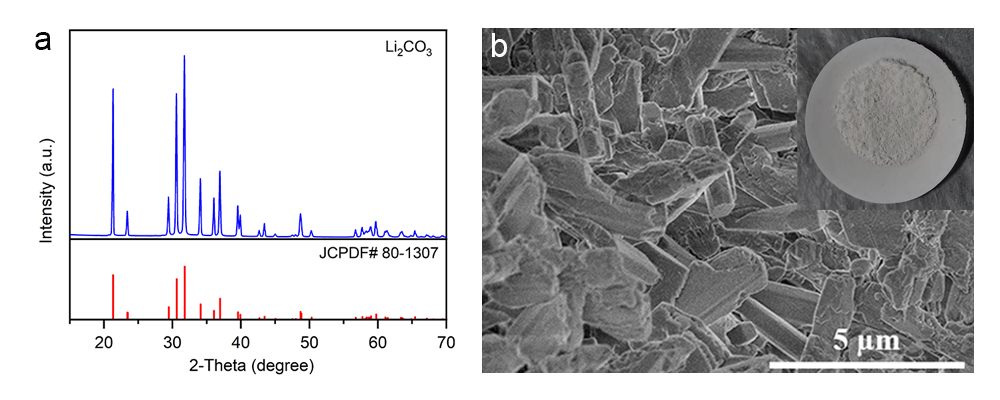


**Fig. S21** XRD pattern (a) and SEM image (b) of Li_2_CO_3_ products, wherein the inset is the photograph of Li_2_CO_3_ powder on filter paper.

**Reference:**

1 M. Weiss, D. A. Weber, A. Senyshyn, J. Janek and W. G. Zeier, Correlating Transport and Structural Properties in Li_1+x_Al_x_Ge_2–x_(PO_4_)_3_ (LAGP) Prepared from Aqueous Solution, *ACS Appl. Mater.,* 2018, **10**, 10935-10944.

2 V. M. Zallocco, J. M. Freitas, N. Bocchi and A. C. M. Rodrigues, Electrochemical stability of a NASICON solid electrolyte from the lithium aluminum germanium phosphate (LAGP) series, *Solid State Ion.,* 2022, **378**, 115888.

3 T. Xu, B. Wu, L. Hou, Y. Zhu, F. Sheng, Z. Zhao, Y. Dong, J. Liu, B. Ye, X. Li, L. Ge, H. Wang and T. Xu, Highly Ion-Permselective Porous Organic Cage Membranes with Hierarchical Channels, *J. Am. Chem. Soc.,* 2022, **144**, 10220-10229.

4 F. Sheng, N. U. Afsar, Y. Zhu, L. Ge and T. Xu, PVA-Based Mixed Matrix Membranes Comprising ZSM-5 for Cations Separation, *Membranes,* 2020, **10**, 114.

5 G. Kresse and J. Hafner, Ab initio molecular-dynamics simulation of the liquid-metal--amorphous-semiconductor transition in germanium, *Phys. Rev. B,* 1994, **49**, 14251-14269.

6 G. Kresse and J. Furthmüller, Efficient iterative schemes for ab initio total-energy calculations using a plane-wave basis set, *Phys. Rev. B,* 1996, **54**, 11169-11186.

7 D. S. Sholl and R. P. Lively, Seven chemical separations to change the world, *Nature,* 2016, **532**, 435-437.

8 J. P. Perdew, K. Burke and M. Ernzerhof, Generalized Gradient Approximation Made Simple, *Phys. Rev. Lett.,* 1996, **77**, 3865-3868.

9 J. Kang, H. Chung, C. Doh, B. Kang and B. Han, Integrated study of first principles calculations and experimental measurements for Li-ionic conductivity in Al-doped solid-state LiGe_2_(PO_4_)_3_ electrolyte, *J. Power Sources,* 2015, **293**, 11-16.

10 Y. Sun, Q. Wang, Y. Wang, R. Yun and X. Xiang, Recent advances in magnesium/lithium separation and lithium extraction technologies from salt lake brine, *Sep. Purif. Technol.,* 2021, **256**, 117807.

11 X. Zhang, Z. Li, J. Liu, F. Xu, L. Zheng, S. De Wolf, Z. Lai and X. Lu, Solar-driven ultrafast lithium extraction from low-grade brine using microfluidics-mediated vortex in scalable electrochemical reactors, *Chem. Eng. J.,* 2023, **454**, 140074.

12 C. Zhang, Y. Mu, S. Zhao, W. Zhang and Y. Wang, Lithium extraction from synthetic brine with high Mg^2+^/Li^+^ ratio using the polymer inclusion membrane, *Desalination,* 2020, **496**, 114710.

13 L. Xu, X. Zeng, Q. He, T. Deng, C. Zhang and W. Zhang, Stable ionic liquid-based polymer inclusion membranes for lithium and magnesium separation, *Sep. Purif. Technol.,* 2022, **288**, 120626.

14 T. Xu, M. A. Shehzad, X. Wang, B. Wu, L. Ge and T. Xu, Engineering Leaf-Like UiO-66-SO_3_H Membranes for Selective Transport of Cations, *Nano-Micro Lett.,* 2020, **12**, 51.

15 N. U. Afsar, M. A. Shehzad, M. Irfan, K. Emmanuel, F. Sheng, T. Xu, X. Ren, L. Ge and T. Xu, Cation exchange membrane integrated with cationic and anionic layers for selective ion separation via electrodialysis, *Desalination,* 2019, **458**, 25-33.

16 Z. Lu, Y. Wu, L. Ding, Y. Wei and H. Wang, A Lamellar MXene (Ti_3_C_2_T_x_)/PSS Composite Membrane for Fast and Selective Lithium-Ion Separation, *Angew. Chem. Int. Ed.,* 2021, **60**, 22265-22269.

17 F. Sheng, B. Wu, X. Li, T. Xu, M. A. Shehzad, X. Wang, L. Ge, H. Wang and T. Xu, Efficient Ion Sieving in Covalent Organic Framework Membranes with Sub-2-Nanometer Channels, *Adv. Mater.,* 2021, **33**, 2104404.

18 R. Xu, Y. Kang, W. Zhang, B. Pan and X. Zhang, Two-dimensional MXene membranes with biomimetic sub-nanochannels for enhanced cation sieving, *Nat. Commun.,* 2023, **14**, 4907.

19 Y. Guo, Y. Ying, Y. Mao, X. Peng and B. Chen, Polystyrene Sulfonate Threaded through a Metal-Organic Framework Membrane for Fast and Selective Lithium-Ion Separation, *Angew. Chem. Int. Ed.,* 2016, **55**, 15120-15124.

20 T. Xu, M. A. Shehzad, D. Yu, Q. Li, B. Wu, X. Ren, L. Ge and T. Xu, Highly Cation Permselective Metal–Organic Framework Membranes with Leaf-Like Morphology, *ChemSusChem,* 2019, **12**, 2593-2597.

21 T. Xu, F. Sheng, B. Wu, M. A. Shehzad, A. Yasmin, X. Wang, Y. He, L. Ge, X. Zheng and T. Xu, Ti-exchanged UiO-66-NH_2_–containing polyamide membranes with remarkable cation permselectivity, *J. Membr. Sci.,* 2020, **615**, 118608.

22 N. Ul Afsar, X. Ge, Z. Zhao, A. Hussain, Y. He, L. Ge and T. Xu, Zwitterion membranes for selective cation separation via electrodialysis, *Sep. Purif. Technol.,* 2021, **254**, 117619.

23 W. Wang, Y. Zhang, X. Yang, H. Sun, Y. Wu and L. Shao, Monovalent Cation Exchange Membranes with Janus Charged Structure for Ion Separation, *Engineering,* 2022.

24 S. J. Warnock, R. Sujanani, E. S. Zofchak, S. Zhao, T. J. Dilenschneider, K. G. Hanson, S. Mukherjee, V. Ganesan, B. D. Freeman, M. M. Abu-Omar and C. M. Bates, Engineering Li/Na selectivity in 12-Crown-4–functionalized polymer membranes, *Proc. Natl. Acad. Sci.,* 2021, **118**, e2022197118.
